# RapidAIM: a culture- and metaproteomics-based Rapid Assay of Individual Microbiome responses to drugs

**DOI:** 10.1186/s40168-020-00806-z

**Published:** 2020-03-11

**Authors:** Leyuan Li, Zhibin Ning, Xu Zhang, Janice Mayne, Kai Cheng, Alain Stintzi, Daniel Figeys

**Affiliations:** 1 Department of Biochemistry, Microbiology and Immunology, Ottawa Institute of Systems Biology, Faculty of Medicine, University of Ottawa, Ottawa, Canada; 2 Canadian Institute for Advanced Research, Toronto, Canada

**Keywords:** Gut microbiome, Drug response, In vitro culturing, Metaproteomics, Absolute abundance, Functional profile

## Abstract

**Background:**

Human-targeted drugs may exert off-target effects or can be repurposed to modulate the gut microbiota. However, our understanding of such effects is limited due to a lack of rapid and scalable assay to comprehensively assess microbiome responses to drugs. Drugs and other compounds can drastically change the overall abundance, taxonomic composition, and functions of a gut microbiome.

**Results:**

Here, we developed an approach to screen compounds against individual microbiomes in vitro, using metaproteomics to both measure absolute bacterial abundances and to functionally profile the microbiome. Our approach was evaluated by testing 43 compounds (including 4 antibiotics) against 5 individual microbiomes. The method generated technically highly reproducible readouts, including changes of overall microbiome abundance, microbiome composition, and functional pathways. Results show that besides the antibiotics, the compounds berberine and ibuprofen inhibited the accumulation of biomass during in vitro growth of the microbiota. By comparing genus and species level-biomass contributions, selective antibacterial-like activities were found with 35 of the 39 non-antibiotic compounds. Seven of the compounds led to a global alteration of the metaproteome, with apparent compound-specific patterns of functional responses. The taxonomic distributions of altered proteins varied among drugs, i.e., different drugs affect functions of different members of the microbiome. We also showed that bacterial function can shift in response to drugs without a change in the abundance of the bacteria.

**Conclusions:**

Current drug-microbiome interaction studies largely focus on relative microbiome composition and microbial drug metabolism. In contrast, our workflow enables multiple insights into microbiome absolute abundance and functional responses to drugs. The workflow is robust, reproducible, and quantitative and is scalable for personalized high-throughput drug screening applications.

## Background

Human-targeted drugs are primarily developed for their effects on the host, and little is known on their effects on the microbiome. Microbiome response to drugs could contribute to off-target drug effect [1]. In addition, the gut microbiome has been linked to gastroenterological, neurologic, respiratory, metabolic, hepatic, and cardiovascular diseases [2]. Therefore, targeting the microbiome could lead to novel therapies [3]. Orally administrated drugs go through complex processing by the host [4], with some drugs reaching the microbiome. As well, drugs can be repurposed to target the microbiome using enteric/colonic delivery approaches [5]. Although the effects of some drugs and compounds on the microbiome have been reported [6], many drug-microbiome interactions are unknown. This is due in part to the extremely high numbers of marketed drugs [7] and compounds in development [8] together with the lack of assays that can rapidly and comprehensively assess the effects of compounds on individual microbiomes.

Different in vitro approaches have been employed to study drug-microbiome interactions. One strategy involves long-term stabilization of the microbiota, as shown in various intestinal microbiota simulators based on continuous flow [9–11]. This approach typically requires a long culture period to stabilize the microbiota (15–20 days), and notable shifts in taxonomic compositions compared with the inoculum have been shown [9, 12]. Moreover, the size and complexity of these culturing systems limit the number of individual microbiomes and drugs that can be examined [11], and thus may not be suitable for high-throughput drug screening purpose. Another strategy is to culture individual bacteria strains isolated from microbiomes. A recent study examined the effects of approved drugs on the biomass of forty individually cultured bacterial strains in a high-throughput manner [13]. This approach highlighted the importance of biomass in identifying antibacterial effects. However, it did not take into account the complexity of a microbial community that could lead to different microbial responses. Approaches such as optical density measurement [13], flow cytometry [14], and quantitative real-time PCR [15] can be used to compare microbiome biomass. However, these approaches lack insights into drug impacts on microbial composition and functions, which are highly related to healthy and disease states. There has been no report of an in vitro gut microbiome-based drug screening approach that could assess both biomass responses and functional alterations in a single analytical test.

The development of meta-omics approaches allowed rapid and deep measurement of microbiome compositions and functional activities. Genetic approaches such as sequencing of 16S rRNA gene fragment amplicons and shotgun metagenomics have been regarded as the “gold standard” in microbiome analysis, providing relative quantifications of microbiome composition and functional capabilities [16, 17]. Notably, different microbial members can differ by several orders of magnitude in biomass [18]. Moreover, there is little insight on which microbial traits actually contribute to the functional activities of the microbiome, as functions predicted from metagenomics analyses are not necessarily expressed. Studies have shown that gene copy numbers are not representative of protein levels [19]. In addition, RNA expression have limited correlation to the actual protein abundance [20]. In contrast, mass spectrometry (MS)-based metaproteomics technology allows for deep insight into proteome-level information of the microbiome [21, 22], providing quantified protein abundances that estimate the functional activities of the microbiome. Proteins not only provide the biological activities to the microbiome but also contribute the majority of biomass in microbial cells. Hence, the metaproteomic readouts can also be used to assess the microbiome biomass and analyze community structure [23]. Metaproteomics has been previously validated to estimate the microbiome and individual microbe biomasses [24], and it readily quantifies the bacterial species responsible for > 90% of the total microbiome biomass [25], making it sufficient for a fast-pass drug screening application.

Here, we report an approach named Rapid Assay of Individual Microbiome (RapidAIM), which applies metaproteomics to gain insights into the microbiome responses to drugs in an in vitro model [26]. Forty-three compounds that have been previously suggested to impact, interact with, or be metabolized by the gut microbiome were selected for this study (Supplementary Table S1). Briefly, in RapidAIM, individual microbiomes are cultured in a previously optimized culture system [26] for 24 h, and the samples are then analyzed using a metaproteomics-based analytical approach. A high-throughput equal-volume based protein extraction and digestion workflow was applied to enable absolute biomass assessment along with the functional profiling. To demonstrate the feasibility and performance of the RapidAIM assay, we carried out a proof-of-concept study involving the 43 compounds and 5 individual gut microbiomes. Microbiome responses including changes in biomass, taxon-specific biomass contributions, taxon-specific functional activities, and detailed responses of specific enzymatic pathways can be obtained following the assay.

## Results

### Development and evaluation of RapidAIM

**Fig. 1:**
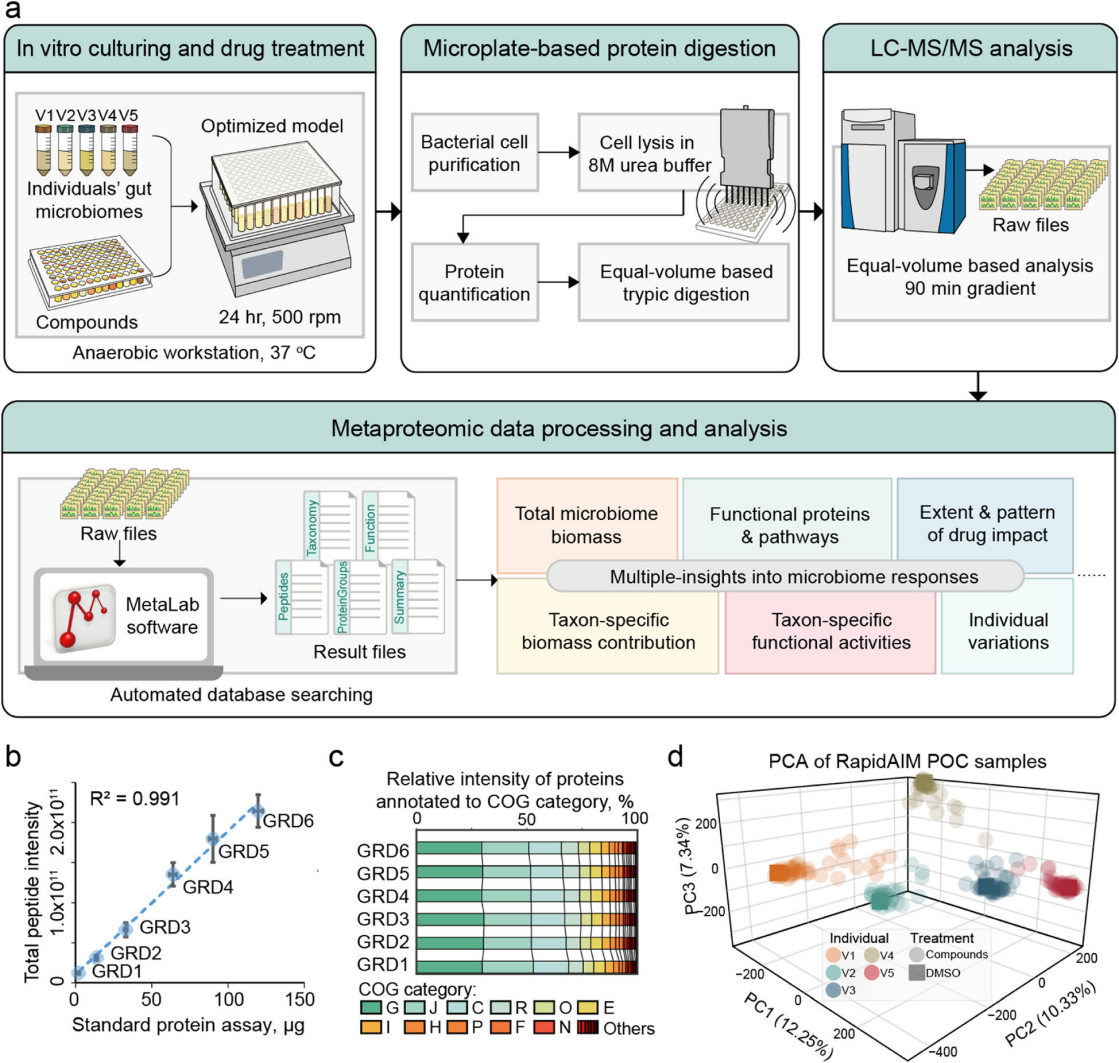
Rapid Assay of Individual Microbiome (RapidAIM) workflow and performance. **a** Experimental, analytical, and bioinformatics components of the RapidAIM workflow. Each individual’s gut microbiome samples are cultured with the test compounds in a 96-well deep-well plate at 37 °C in strict anaerobic conditions for 24 h followed by high-throughput sample preparation and rapid LC-MS/MS analysis. Peptide and protein identification and quantification, taxonomic profiling, and functional annotation were performed using the automated MetaLab software [27]. **b** A series of six dilutions (dilution gradients: GRD1~6) of a same microbiome sample was tested in triplicates through the equal-volume digestion and equal-volume MS loading protocol; the summed peptide intensity was compared with a set of protein concentration standards provided with DC protein concentration assay and showed good linearity (center points and error bars represent mean ± SD). **c** Stacked bars of clusters of orthologous groups (COG) category levels across the six concentrations showing no bias at the functional quantifications. **d** PCA based on LFQ intensities of protein groups for all POC samples

RapidAIM consists of an optimized microbiome culturing method, an equal-volume based protein extraction and digestion workflow and a metaproteomic analysis pipeline (Fig. 1a). Briefly, fresh human stool samples are inoculated in 96-well deep-well plates and cultured with drugs for 24 h. We have previously optimized the culture model and validated that it maintains the composition and taxon-specific functional activities of individual gut microbiomes in 96-well plates [26]. After 24 h, the cultured microbiomes are prepared for metaproteomic analysis using a microplate-based metaproteomic sample processing workflow (Supplementary Figure S1) adapted from our single-tube protocol [28]. The microplate-based workflow consists of bacterial cell purification, cell lysis with ultrasonication in 8 M urea buffer, in-solution tryptic digestion, and desalting. We validated each step of this workflow and found no significant differences in identification efficiency between 96-well plate processing and single-tube processing (Supplementary Figure S1). To compare total biomass, taxon-specific biomass and pathway contributions between samples in a high-throughput assay format, we applied an equal sample volume strategy to our recently developed metaproteomics techniques [22, 27, 29]. To validate the absolute quantification of microbiome abundance by comparing total peptide intensity, an equal volume of samples from a microbiome dilution series (simulating different levels >of drug effects) was taken for tryptic digestion and LC-MS/MS analysis. Summed peptide intensity in each sample showed good linearity (*R*^2^ = 0.991, Fig. 1b) with a standard colorimetric protein assay, showing that the total peptide intensity is a good indicator for microbiome biomass levels. Because drugs can cause drastic changes in microbiome abundance, we then evaluated whether biomass differences between wells could also cause bias in identified functional and taxonomic compositions. We confirmed that the level of total biomass did not bias the composition of functional profiles (Fig. 1c), protein groups (Supplementary Figure S2a), or taxonomic abundances (Supplementary Figure S2b).

### RapidAIM: proof-of-concept study

We conducted a proof-of-concept (POC) study on the use of RapidAIM to characterize drug effect on the microbiome. We selected 43 compounds that have been previously suggested to impact, interact with, or be metabolized by the gut microbiome (Supplementary Table S1). Thirty-seven of these compounds are FDA-approved drugs; 4 are antibiotics, and the others include nonsteroidal anti-inflammatory drugs (NSAIDs), anti-diabetic drugs, aminosalicylate, and statins. Each compound, at a concentration corresponding to the maximal daily oral dose distributed in 200 g daily fecal wet weight [30], was added to 5 wells of 96-well plates containing 1-mL culture medium in each well. The drug solvent, dimethyl sulfoxide (DMSO), was used as the negative control. Then, each of the 5 wells for each compound was inoculated with a different fecal microbiome from healthy human volunteers. Following 24 h of culturing, the samples were processed through the microplate-based workflow (Supplementary Figure S1) and were subjected to a 90 min gradient-based rapid LC-MS/MS analysis. Using our automated metaproteomic data analysis software MetaLab [27], 101,995 peptide sequences corresponding to 24,631 protein groups were quantified across all samples with a false discovery rate (FDR) threshold of 1% (Supplementary Figure S3a). The average MS/MS identification rate was 32.4 ± 8.8% (mean ± SD); an average of 15,017 ± 3654 unique peptides and 6684 ± 998 protein groups were identified per sample (Supplementary Figure S3c-d). To provide a global overview of the microbiome responses, a PCA was performed based on label-free quantification (LFQ) intensities of protein groups (Fig. 1d). As expected, the samples clustered based on the original microbiome source and not based on drug treatment. A PerMANOVA test [31] based on Bray-Curtis dissimilarities [32, 33] showed that the samples were significantly clustered according to different individuals (*p* = 0.001 based on 999 permutations). Within each individual microbiome group, a number of drug-treated samples clustered closely to their control, while several other samples clearly separated from the non-treated control.

We next evaluated the robustness and reproducibility of the method by culturing one microbiota with drugs in technical triplicates. Cultured triplicates yielded high Pearson’s *r* for LFQ protein group intensities (Supplementary Figure S3b). Hierarchical clustering based on Pearson’s *r* of LFQ protein group intensities between samples showed that with the exception of several compounds which clustered closely with DMSO; cultured triplicates were clustered together (Supplementary Figure S4a). Moreover, total biomass, functional enzymes, and species biomass contributions were highly reproducible between triplicates, as shown in Supplementary Figure S4b-d.

### Effects of compounds on microbiome abundance and composition

**Fig. 2:**
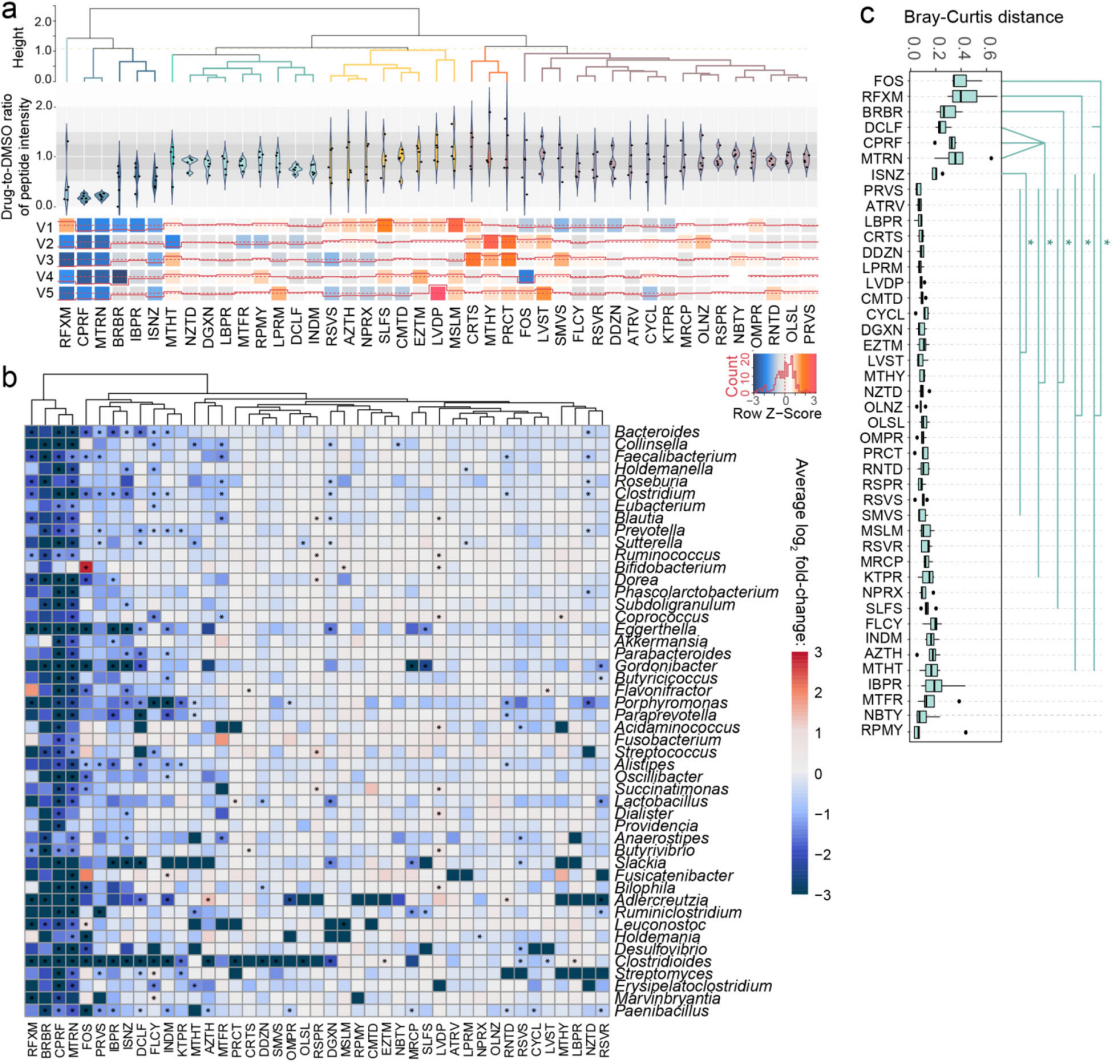
Response of microbiome abundance and composition to compounds. **a** Biomass responses of individuals’ microbiomes to compounds relative to DMSO control. Ratio of peptide intensity between compound and DMSO control samples was calculated for each individual microbiome. **b** Log_2_ fold-change of absolute abundance at the genus level in response to each drug compared with the DMSO control. Genera that existed in ≥ 80% of the volunteers are shown. Asterisk indicates significantly changed bacterial abundance by Wilcoxon test, *p* < 0.05. **c** Bray-Curtis distance of genus-level composition between drug-treated microbiomes and the corresponding DMSO control samples. Heatmap colors are generated with average of log_2_ fold-changes among the five individual samples. Statistical significance was calculated by pairwise Wilcoxon test (FDR-adjusted *p* < 0.05). Box spans interquartile range (25th to 75th percentile), and line within box denotes median. For full compound names, see abbreviation list in Supplementary Table S1

We examined the effect of the 43 compounds on the overall abundance (biomass) of each individual microbiome by comparing the total peptide intensity (Fig. 2a). As expected, the antibiotics greatly reduced total microbial biomass in most individual microbiomes (with one exception of increased microbiome abundance in response to rifaximin, further examination is shown in Supplementary Figure S5). Similar to these antibiotics, berberine and ibuprofen also inhibited the biomass of all individual microbiomes.

We next explored the effects of drugs on the microbiome composition based on bacterial biomass contributions. To evaluate the overall shift of the microbiome, Bray-Curtis distance [32, 33] between drug-treated and DMSO control microbiome indicated that fructooligosaccharide (FOS), rifaximin, berberine, diclofenac, ciprofloxacin, metronidazole, and isoniazid significantly shifted the microbiome (pairwise Wilcoxon test, FDR-adjusted *p* < 0.05; Fig. 2c).

We further examined the response of individual bacterial taxon using their absolute abundance estimated using the sum of all peptide intensities for each taxon as previously described [27] (Fig. 2b). In contrast to genetic sequencing-based approaches, which often only report relative abundance, metaproteomics measure absolute abundances. As expected, the broad-spectrum antibiotics rifaximin, ciprofloxacin, and metronidazole significantly reduced the total biomass (Fig. 2a) and the absolute abundance of a many bacterial genera (Fig. 2b, Wilcoxon test, *p* < 0.05). Nevertheless, ciprofloxacin and metronidazole significantly increased the relative abundance of genera *Bifidobacterium*, *Ruminococcus*, *Butyrivibrio*, *Paenibacillus*, etc. (Supplementary Figure S6). Non-antibiotic compounds, such as berberine, FOS, pravastatin, ibuprofen, diclofenac, flucytosine, and indomethacin also showed significant decreases in the abundances of over 10 genera. In addition, selective antibacterial activities were found in 35 out of the 39 non-antibiotic compounds at the genus level; at the species level, we found that 32 non-antibiotic compounds significantly altered the biomass of at least one bacterial species (one-sided Wilcoxon rank sum test, FDR-adjusted *p* < 0.05; Supplementary Table S2). Interestingly, members of the Actinobacteria phyla, including *Eggerthella*, *Gordonibacter*, *Slackia*, and *Adlercreutzia*, were the most susceptible to drugs compared with most other genera (Supplementary Figure S6). In summary, RapidAIM allowed for the assessment of changes in both absolute and relative abundances of microbes in response to compounds.

### Overall functional profiles in response to compounds

**Fig. 3:**
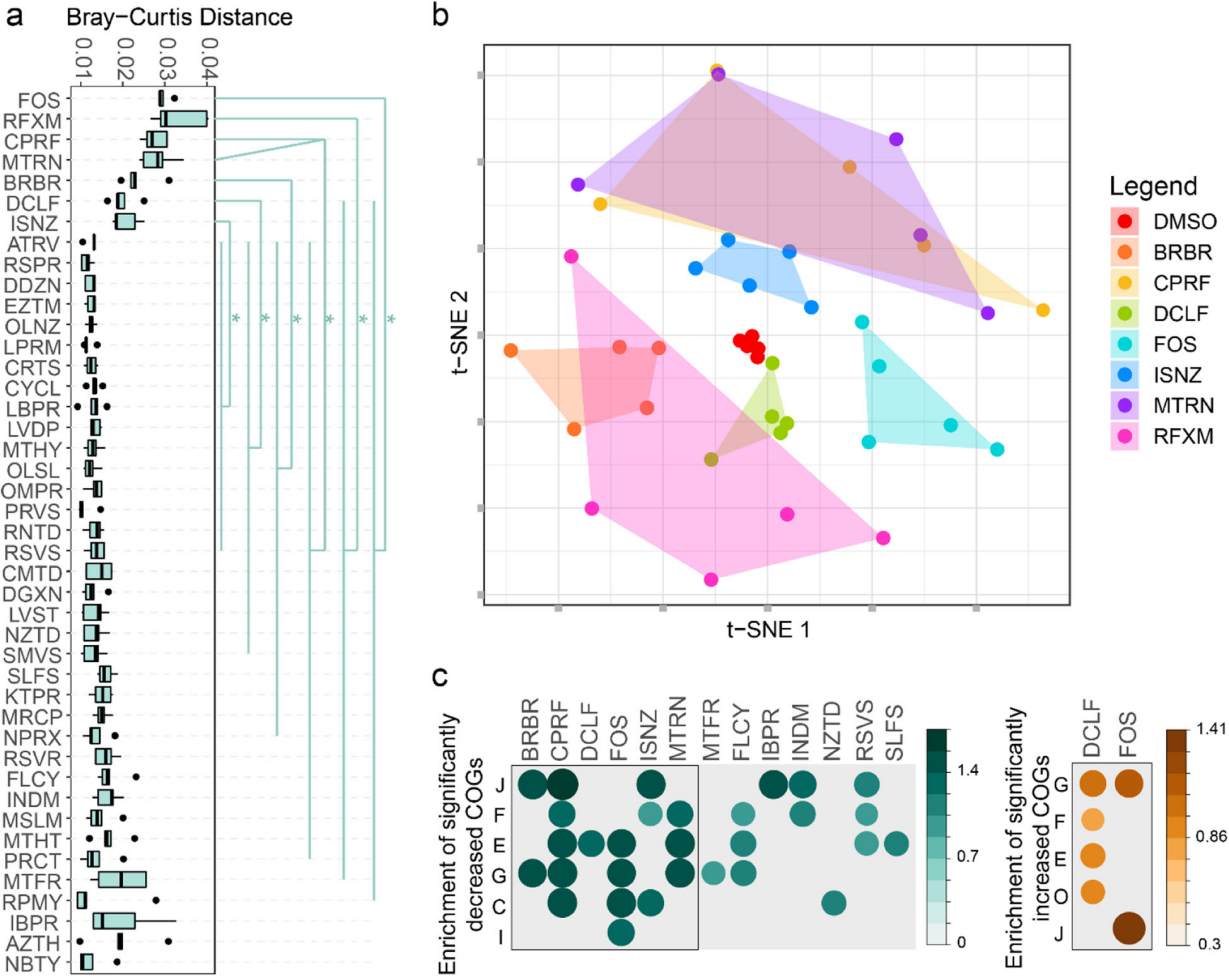
Effect of compounds on metaproteomic profiles of the microbiome. **a** Bray-Curtis distance of protein groups between drug-treated microbiomes and the corresponding DMSO control samples. Statistical significance was calculated by pairwise Wilcoxon test (FDR-adjusted *p* < 0.05). **b** Unsupervised dimensionality reduction analysis showing compound-specific patterns of responses to class I compounds. **c** Enrichment analysis of all significantly different COGs in the POC dataset. Significantly altered COGs with a *p* value cutoff of 0.05 are shown

The Bray-Curtis distance of protein group profiles showed that all the four antibiotics, as well as FOS, berberine, and diclofenac, significantly altered the microbiome functions (Fig. 3a). These functional alterations likely stemmed from changes in taxonomic composition as revealed by the genus-level Bray-Curtis distance analysis (Fig. 2c**)**. We next analyzed the protein group intensities by partial least square discriminant analysis (PLS-DA) to determine whether metaproteomic profiles could be used to discriminate between the DMSO control and each of the drug-treated microbiomes. In agreement with the Bray-Curtis analysis results, PLS-DA interpretation identified drug-specific metaproteomic patterns associated with the seven abovementioned compounds, i.e., the four antibiotics, FOS, berberine, and diclofenac (Supplementary Figure S7). Hence, hereafter, we named these seven compounds as class I compounds, whereas others were named class II compounds. To gain a better understanding of the global effects of class I compounds on the gut microbiome, we applied an unsupervised non-linear dimensionality reduction algorithm, t-distributed stochastic neighbor embedding (t-SNE) [34], to visualize this subgroup of metaproteomic data based on protein group abundances (Fig. 3b). Class I compounds led to a global alteration of the metaproteome, with apparent compound-specific patterns.

We next examined the drug impacts on the abundance of functional proteins according to clusters of orthologous groups (COG) of proteins. We identified 535 COGs significantly decreased by at least 1 drug treatment; 15 of these COGs were decreased by ≥ 10 compounds (Supplementary Figure S8). Diclofenac and FOS were the only 2 compounds that significantly increased COGs (55 and 81 COGs, respectively). Enrichment analysis based on these significantly altered COGs shows that COG categories found to be enriched were responsive to 13 of our compounds (Fig. 3c), 6 of those were class I compounds. Interestingly, the non-antibiotic NSAID diclofenac increased the abundance of several COG categories (Fig. 3c). By mapping these significantly increased proteins from these COG categories against the string database, we found that these altered proteins are functionally interconnected (Supplementary Figure S9).

### Specific proteins and enzymatic pathways in response to compounds

**Fig. 4:**
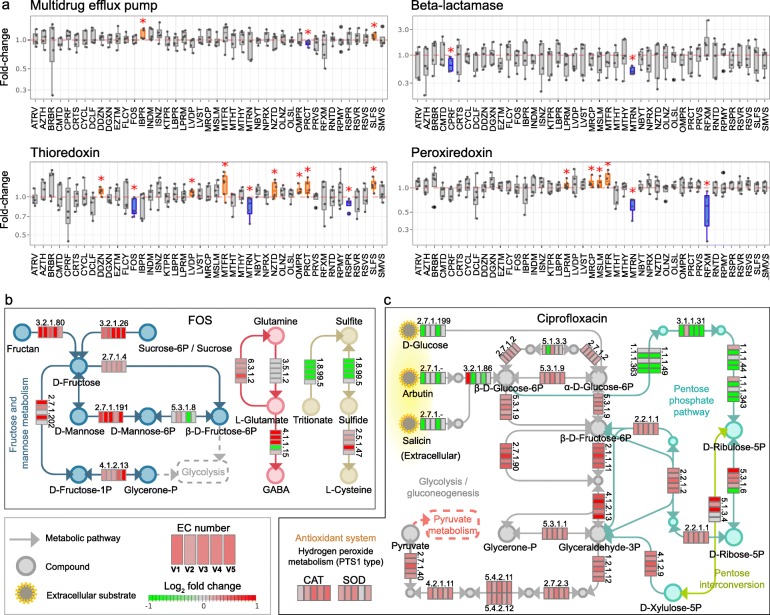
Response of specific proteins and enzymatic pathways to drug treatment. **a** Response of proteins related to drug resistance (red asterisk denotes *p* < 0.05 by Wilcoxon test); boxplots of all functional responses of the RapidAIM POC dataset are available at https://shiny.imetalab.ca/RapidAIM_functional_response/. **b** Effect of FOS treatment on enzymes involved in fructose and mannose metabolism, GABA production, and sulfide metabolism pathways. **c** Effect of ciprofloxacin treatment on enzymes involved in the glycolysis/gluconeogenesis and pentose phosphate pathway. The five blocks of each enzyme represent the five individual microbiomes. Colors in the blocks represent differences between normalized KEGG enzyme intensities with drug treatment versus DMSO (log_2_ transformation of the original intensity followed by a quotient normalization (*x*/mean))

Next, we examined the ability of RapidAIM to observe the modulations of specific proteins and enzymatic pathways of interest. As an example, we show that proteins related to drug resistance were significantly altered by several compounds (Wilcoxon test, Fig. 4a). Multidrug efflux pump proteins that could extrude structurally dissimilar organic compounds [35] were significantly increased by ibuprofen and sulfasalazine treatment. Antibiotic-degrading enzyme beta-lactamase [36] was significantly decreased by the antibiotics ciprofloxacin and metronidazole. Thioredoxin participates in defense against oxidative stress induced by drugs via peroxiredoxin [37]. Significant increases in thioredoxin and/or peroxiredoxin were observed in ten of our treatments. In addition, we analyzed the response of enzymes along the butyrate production pathway. In response to the antifungal drug flucytosine, five out of seven observed enzymes showed significant decreases (Supplementary Figure S10). As another group of examples, we show the effects of FOS and ciprofloxacin on specific enzymatic pathways in an individual microbiome view (Fig. 4b, c). Protein groups were annotated to KEGG (Kyoto Encyclopedia of Genes and Genomes) enzymes and were mapped against the KEGG pathway database. FOS increased enzymes responsible for fructan and sucrose uptake, as well as enzymes for conversion of d-fructose into d-fructose-1-phosphate, d-mannose-6-phosephate, and β-d-fructose-6-phosphate (Fig. 4b). FOS also affected enzymes involved in the interconversion between glutamine, glutamate, and GABA (molecules involved in gut-brain communication). In addition, enzymes involved in sulfide accumulation were affected, including decrease of dissimilatory sulfite reductase (EC 1.8.99.5) and increase of cysteine synthase (EC 2.5.1.47) (Fig. 4b). Ciprofloxacin significantly altered the levels of enzymes involved in glycolysis/glycogenesis and pentose phosphate pathways (Fig. 4c). The majority of enzymes involved in glycolysis were significantly increased by ciprofloxacin. Ciprofloxacin down-regulated enzymes (ECs 1.1.1.49/1.1.1.363, 3.1.1.31, 1.1.1.44/1.1.1.343) involved in synthesis of ribulose-5-phosphate, which can be isomerized to ribose 5-phosphate for nucleotide biosynthesis [38]. Moreover, the levels of antioxidant enzymes superoxide dismutase (SOD) and catalase (CAT) were increased, suggesting that ciprofloxacin induces oxidative stress in gut bacteria (Fig. 4c).

### Taxon-specific functional responses to class I compounds

**Fig. 5:**
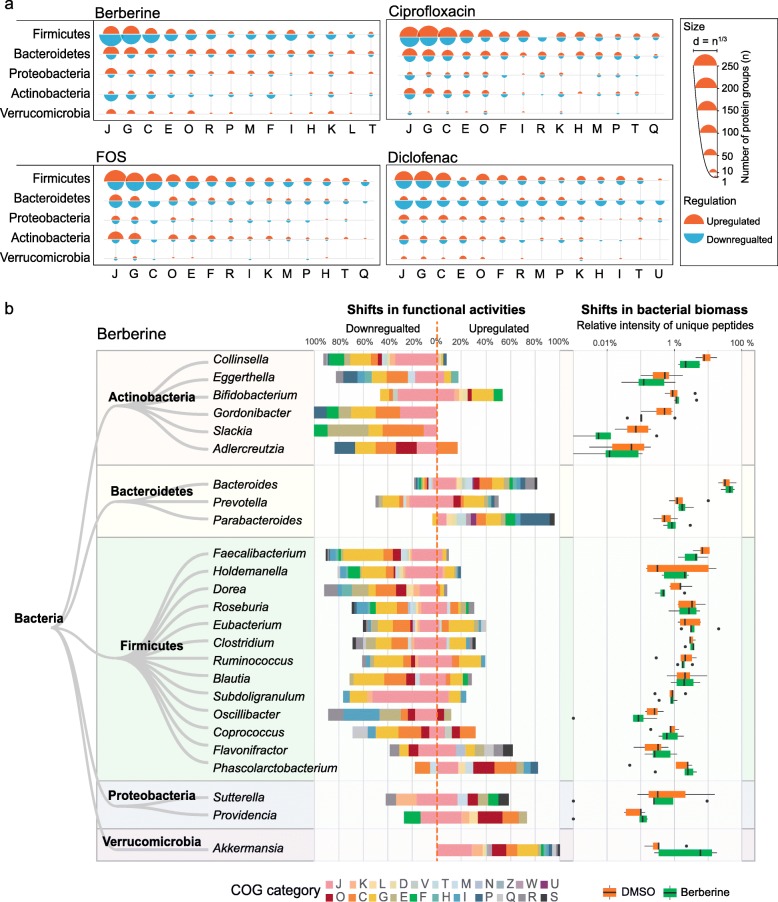
Global functional effects of berberine, ciprofloxacin, FOS and diclofenac. **a** Taxon-function distribution of protein groups responding to berberine, FOS, ciprofloxacin, and diclofenac. Responding protein groups were selected by PLS-DA based on ComBat-corrected data. The semicircle diameter represents the number of PLS-DA VIP > 1 protein groups corresponding to each phyla-COG category pair. **b** Genus level shifts in functional activities in response to berberine and the alterations in biomass of the corresponding genera. Functional shifts (differential protein groups) were identified by PLS-DA. For each genus, the percentages of the total numbers of up- and downregulated protein groups corresponding to each COG category are shown. Shifts in bacterial biomass in the five microbiomes are shown in box plots with the boxes spanning interquartile range (25th to 75th percentile) and the vertical lines denoting the median for each genus

We next performed a taxonomic analysis correlating to the functional responses to diclofenac, FOS, ciprofloxacin, and berberine, which represent four different types of compounds (NSAID, oligosaccharide, antibiotics, anti-diabetes) in the class I. Protein groups with VIP scores > 1 (thereafter defined as differential proteins) were extracted from each model and were annotated with their taxonomic and COG information. The taxonomic distributions of the differential proteins varied among drugs (Fig. 5a). Moreover, mapping of the differential proteins to phyla-specific pathways revealed phyla-specific responses, as shown for berberine in Supplementary Figure S11. In agreement with Fig. 5a, proteins with decreased abundance had a higher pathway coverage than the proteins increased in Firmicutes and Actinobacteria, while the opposite pattern was observed in Bacteroidetes, Proteobacteria, and Verrucomicrobia. In some cases, the phylum-specific responses included both increased and decreased proteins within the same pathway (black lines, Supplementary Figure S11). For example, we observed this pattern in fatty acid, carbohydrate, and nucleotide metabolism pathways in Firmicutes.

Genus-level analysis revealed genus-specific responses to berberine (Fig. 5b). In most genera, the genus-specific responses correlated with the overall abundance of the corresponding genus (Fig. 5b, right panel). Nevertheless, some genera showed functional shifts in response to berberine without changes in overall abundance. For example, *Bifidobacteria*, *Roseburia*, *Eubacterium*, *Clostridium*, *Ruminococcus*, *Blautia*, and *Subdoligranulum* exhibited downregulation of proteins in various COG categories but no changes in biomass were observed.

### Gut microbiome functions altered by class II compounds

**Fig. 6:**
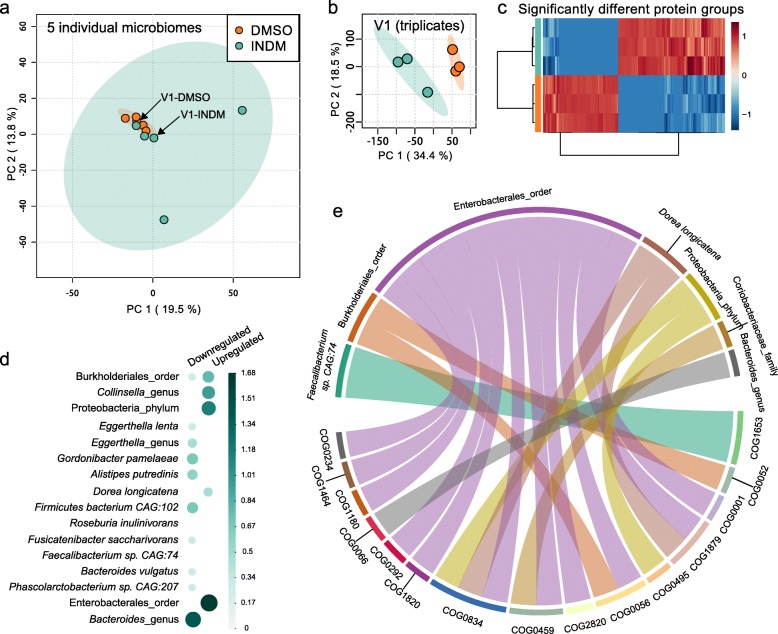
Individual functional responses to indomethacin. **a** When visualizing the responses of several individual microbiomes with PCA (based on LFQ intensities of protein groups), inter-individual variability can be greater than drug-induced functional shifts. **b** PCA clearly differentiated the response of microbiome V1 treated in triplicates using RapidAIM. **c** Three hundred three significant protein group responders were found by *t* test (FDR-adjusted *p* < 0.05). **d** Taxon enrichment analysis based on the differential protein groups, (*p*-adjusted = 0.05). **e** Taxon-function coupled enrichment analysis of upregulated protein groups

Class II compounds, in contrast to class I compounds, did not cause a global shift in the five individual microbiomes (an example is given by indomethacin, Fig. 6a). However, Fig. 6a as well as the Bray-Curtis analyses (Figs. 2c and 3a) suggested that there was individual variability in the extent of drug response. We show that when analyzed on an individual subject basis, significant individualized functional effects are present. For example, PCA showed a clear differentiation between indomethacin-treated microbiome and the DMSO control in cultured replicates of a single indomethacin-treated microbiota from individual V1 (Fig. 6b), where, 303 significantly altered protein groups were identified (Fig. 6c). These protein expression shifts suggest high sensitivity of the RapidAIM assay in its application to personalized drug screenings. Taxon-function coupled enrichment analysis showed that protein groups with decreased abundances were highly enriched in the genus *Bacteroides*. In addition, increased protein groups were also enriched in *Gordonibacter pamelaeae*, *Firmicutes bacterium* CAG:102, *Alistipes putredinis*, *Eggerthella* genus, etc. (Fig. 6d). Protein groups with increased abundances were mostly enriched in the order Enterobacterales and were also enriched in Burkholderiales order, Collinsella genus, and Proteobacteria phylum (Fig. 6d, e). We further analyzed the functional enrichment corresponding to the increased protein groups. Most of the enriched functions corresponded to Enterobacterales. These enriched functions included molecular chaperones COG0459 chaperonin GroEL (HSP60 family) and COG0234 co-chaperonin GroES (HSP10) (Fig. 6e).

## Discussion

In the present study, we developed an approach named RapidAIM to evaluate the effects of xenobiotics on individual microbiomes. The range of xenobiotics that reach the intestine and may interact with the gut microbiome is massive and expanding. These xenobiotics include antibiotics and other pharmaceuticals, phytochemicals, polysaccharides, food additives, and many other compounds. With the exception of antibiotics, we remain surprisingly ignorant on the extent to which these compounds affect the functions of the gut microbiome, and whether these compounds could be repurposed to provide beneficial effects. This understanding was limited by the lack of an efficient and scalable approach that could maximally obtain insights into microbiome responses while minimizing the number of analytical tools being used.

Here, we describe an approach which enables the exploration of drug-microbiome interactions using an optimized in vitro culturing model and a metaproteomic approach. We have achieved the maintenance of the representativeness of the initial individual microbiome [26]. Furthermore, for an in vitro culturing simulating the in vivo microbiome, it is important to note that the population of gut bacteria in the human body is highly dynamic. It has been estimated that there are ~ 0.9·10^11^ bacteria/g wet stool and a total of ~ 3.8·10^13^ bacteria in the colon. Approximately 200-g wet daily stool would be excreted [30], leading to a dramatic decrease of the bacterial number in the gut; on the other hand, new bacterial biomass starts growing on nutrients passing through the gut. Current technologies examining the effect of xenobiotic stimulation are usually based on microbiota stabilized after over 2 weeks of culturing. However, at the stable phase of microbiota growth, the ecosystem reaches its carrying capacity (stable population size), limiting possible observations such as drug effect on the biomass. We have previously validated that the composition of gut microbiota is well maintained along the growth curve using our growth cultures [26], and we were able to observe drug responses of growing gut microbiota by adding the compounds at the initial inoculation stage. Subsequently, combined with our quantitative metaproteomics approach based on equal-sample volume digestion, we were able to observe the drug responses of using the overall microbiome abundance and taxon-specific biomass contributions.

Absolute abundance is a much better measurement of drug effects on the microbiome compared with relative abundance because it details the levels of individual bacteria as well as the summative response at the microbiome level. Moreover, it allows comparisons of effects across multiple drugs. Nevertheless, as the metagenomics field predominantly uses techniques that only report relative abundance, we also provided the relative abundance in Supplementary Figure S6. At first glance, relative and absolute abundance can appear to tell different stories. For example, *Bifidobacterium* is known to be resistant to ciprofloxacin and metronidazole [39] whereas most other genera are not. Therefore, while the total biomass was decreased by ciprofloxacin and metronidazole, the absolute abundance of *Bifidobacterium* remained the same, resulting in an overall increase in its relative abundance. Ibuprofen has been frequently used as a safe medication; however, a recent study suggested that ibuprofen had antimicrobial activity as a side effect [40]. In agreement, we showed that ibuprofen significantly inhibited the overall microbiome biomass, involving decreased absolute abundances of common gut commensals such as *Bacteroides*, *Clostridium*, *Dorea*, *Eggerthella*, and *Akkermansia*. As a final example, previous studies suggested that berberine has positive effect on beneficial gut microbes, e.g., selectively enriched a few putative short-chain fatty acid producing bacteria [41], and increased the relative abundance of *Akkermansia* [42]. However, although we also observed an increase in *Akkermansia*’s relative abundance, its absolute abundance was not affected by berberine. In this example, an enrichment of a taxon (increase in relative abundance) does not necessarily relate to its outgrowth. Therefore, we suggest that absolute abundance provides a better evaluation of the changes induced in the microbiome by drug treatments.

We showed that the RapidAIM assay yielded insights into functional responses at multiple levels. Using PLS-DA, we found that berberine, FOS, metronidazole, isoniazid, ciprofloxacin, diclofenac, and rifaximin consistently shifted the metaproteome of the individual gut microbiomes. By annotating the altered proteins at taxonomy, function, and pathway levels, we revealed the actions of the different drugs on the microbiome. For example, FOS treatment elevated enzymes involved in fructan and sucrose uptake, as well as enzymes involved in the interconversion among glutamine, glutamate, and GABA, which are associated with microbiome communication via the gut-brain axis [43]. In agreement, a study has shown that FOS administration increased GABA receptor genes in mice and further exhibited both antidepressant and anxiolytic effects [44]. FOS also decreased proteins involved in sulfide generation, suggesting decreased sulfide accumulation in the microbiome. This observation is in agreement with in vivo studies showing that FOS treatment decreased the concentration of fecal H_2_S [45–47]. Ciprofloxacin treatment increased enzymes SOD and CAT, which was in agreement with several reports indicating that ciprofloxacin triggers oxidative stress in several bacteria [48–50]. With berberine treatment, we showed that taxon-specific functional shifts can occur either with or without a change in the taxon’s biomass. Our analysis on phylum-specific responses showed that within the same phylum, proteins with the same functionality can increase in one species while decreasing in another, suggesting that there can be strong functional redundancy of species within a phylum. These observations highlight the strength of our workflow which enables quantitative metaproteomic profiling of the microbiome. Indeed, current classical sequencing-based approaches (sequencing of 16S rRNA gene fragment amplicons or metagenomics sequencing), which generate relative abundances, would not detect these types of changes. Finally, we showed that although a compound may not show global impacts across the five tested microbiomes, it could result in significant alterations on a single microbiome basis. The example given by indomethacin indicated that the order Enterobacterales was enriched with increased chaperonin GroEL (HSP60 family) and co-chaperonin GroES (HSP10) (Fig. 6e), which have been implicated in infection and diseases pathology [51].

Our workflow still exhibits certain limitations. In particular, MS analysis is a time-consuming process. To this end, a fast-pass screening process should consider using techniques such as tandem mass tags (TMT) [52, 53] to multiplex multiple microbiome samples in one MS analysis. Furthermore, our workflow only measures the direct effects of compounds on the microbiome. In its current implementation, it does not take into account the host effect on the microbiome and/or the effects of drug metabolites produced by the host. Nevertheless, these metabolites could be tested along with the RapidAIM assay by additional analysis. Future efforts could be aimed at incorporating co-culture of host cells/tissue and gut bacteria [54–56] into a high-throughput drug screening process for achieving more comprehensive insights on host-drug-microbiome interaction. Metaproteomics is a tool that is orthogonal to other omics technologies [19]; hence, for the need of deeper investigations, RapidAIM could also be coupled with techniques such as metagenomics or metabolomics for a multiple dimension view of the microbiome interaction with drugs.

## Conclusion

To date, the field of drug-microbiome interactions largely focuses on relative microbiome composition and microbial drug metabolism, with a limited understanding of the effects of pharmaceuticals on the absolute abundance and the function of the gut microbiome. A better understanding of these interactions is essential given that the drug effects on the microbiome biomass and functions may have important health consequences. Our workflow enabled the insights into both absolute abundances and functional responses of the gut microbiome to drugs using metaproteomics as the single analytical tool. We have shown that our workflow is robust, reproducible, and quantitative, and is easily adaptable for high-throughput drug screening applications.

## Methods

### Stool sample preparation

The Research Ethics Board protocol (# 20160585-01H) for stool sample collection was approved by the Ottawa Health Science Network Research Ethics Board at the Ottawa Hospital. Stool samples were obtained from 5 healthy volunteers (age range 27–36 years; 3 males and 2 females). Exclusion criteria were as follows: IBS, IBD, or diabetes diagnosis; antibiotic use or gastroenteritis episode in the last 3 months; use of pro-/pre-biotic, laxative, or anti-diarrheal drugs in the last month; or pregnancy. All volunteers were provided with a stool collection kit, which included a 50-mL Falcon tube containing 15 mL of sterile phosphate-buffered saline (PBS) pre-reduced with 0.1% (w/v) l-cysteine hydrochloride, a 2.5-mL-sterile sampling spoon (Bel-Art, USA), plastic wrap, gloves, and disposal bags. Briefly, each volunteer placed the plastic wrap over a toilet to prevent the stool from contacting water, collected ~ 3 g of stool with the sampling spoon, and dropped the spoon into the prepared 50-mL tube. The sample was immediately weighed by a researcher and transferred into an anaerobic workstation (5% H_2_, 5% CO_2_, and 90% N_2_ at 37 °C), where the tube was uncapped to remove O_2_ before homogenization with a vortex mixer. Then, the homogenate was filtered using sterile gauzes to remove large particles and obtain the fecal inoculum.

### Culturing of microbiota with drug treatments

Each fecal inoculum was immediately inoculated at a concentration of 2% (w/v) into a 96-well deep-well plate containing 1-mL culture medium and a compound dissolved in 5 μl DMSO (or 5 μl DMSO as the control) in each well. The culture medium contained 2.0 g L^−1^ peptone water, 2.0 g L^−1^ yeast extract, 0.5 g L^−1^l-cysteine hydrochloride, 2 mL L^−1^ Tween 80, 5 mg L^−1^ hemin, 10 μL L^−1^ vitamin K1, 1.0 g L^−1^ NaCl, 0.4 g L^−1^ K_2_HPO_4_, 0.4 g L^−1^ KH_2_PO_4_, 0.1 g L^−1^ MgSO_4_·7H_2_O, 0.1 g L^−1^ CaCl_2_·2H_2_O, 4.0 g L^−1^ NaHCO_3_, 4.0 g L^−1^ porcine gastric mucin (cat# M1778, Sigma-Aldrich), 0.25 g L^−1^ sodium cholate, and 0.25 g L^−1^ sodium chenodeoxycholate [26]. The culture medium was sterile and had been pre-reduced overnight in an anaerobic workstation. Concentration of each compound was determined based on the assumption that maximal oral dosage of the drug distributed in 200 g average weight of the colon contents. However, several compounds (i.e., cimetidine, ciprofloxacin, flucytosine, mesalamine, metformin, metronidazole, naproxen-sodium, paracetamol, rifaximin, sodium butyrate, and sulfasalazine) exceeded solubility in the given volume of DMSO (5 μl). After confirming that these compounds still showed effect after a 10× dilution (as can be seen from hierarchical clustering in Supplementary Figure S3), the concentrations corresponding to the 1/10 highest oral dosages were used for these compounds. Detailed catalog number and concentration of each compound is listed in Supplementary Table S1. After inoculation, the 96-well deep-well plate was covered with a sterile silicone gel mat with a vent hole for each well made by a sterile syringe needle. Then, the plate was shaken at 500 rpm with a digital shaker (MS3, IKA, Germany) at 37 °C for 24 h in the anaerobic chamber.

### Metaproteomic sample processing and LC-MS/MS analysis

The sample processing was based on a previously reported metaproteomic sample processing workflow [57], we adapted it for 96-well plates (Supplementary Figure S1). Briefly, after culturing for 24 h, each 96-well plate was transferred out of the anaerobic station and was immediately centrifuged at 300*g* at 4 °C for 5 min to remove debris. With all plates sitting on ice, the supernatants were transferred into new 96-well deep-well plates for another two rounds of debris removal at 300*g*. The supernatants were then transferred to a new plate and centrifuged at 2272*g* for 1 h to pellet the microbiota. The supernatant was removed, and the pelleted bacterial cells were washed three times with cold PBS in the same 96-well deep-well plate, pelleting the cells after each wash by a 2272*g* centrifugation for 1 h at 4 °C. The 96-well plate containing harvested bacterial cells was then stored overnight at – 80 °C before bacterial cell lysis and protein extraction. The lysis buffer was freshly prepared, containing 8 M urea in 100 mM Tris-HCl buffer (pH = 8.0), plus Roche PhosSTOP™ and Roche cOmplete™ Mini tablets. Microbial cell pellets were then re-suspended in 150 μl lysis buffer and lysed on ice using a sonicator (Q125 Qsonica, USA) with an 8-tip-horn probe. One hundred percent amplitude was used (i.e., 15.6 watts per sample), and four cycles of 30 s ultrasonication and 30 s cooling down were performed. Protein concentrations of the DMSO control samples were measured in triplicate using a detergent compatible (DC) assay (Bio-Rad, USA). Then, a volume equivalent to the average volume of 50 μg of protein in the DMSO control samples was acquired from each sample and placed into a new 96-well deep-well plate. The samples were reduced and alkylated with 10 mM dithiothreitol (DTT) and 20 mM iodoacetamide (IAA), followed by a 10× dilution using 100 mM Tris-HCl (pH = 8.0) and tryptic digestion at 37 °C for 18 h using 1 μg of trypsin per well (Worthington Biochemical Corp., Lakewood, NJ). Digested peptides were desalted using a panel of lab-made 96-channel filter tips generated by inserting 96 20 μl filter tips into a 96-well cover mat and stacking each filter tip with 5 mg of 10-μm C18 column beads. After being washed twice with 0.1% formic acid (v/v), tryptic peptides were eluted with 80% acetonitrile (v/v)/0.1% formic acid (v/v).

After freeze-drying, each sample was re-dissolved in 100 μl 0.1% formic acid (v/v), and 2 μl of the solution (corresponding to 1 μg of proteins in the DMSO control) was loaded for LC-MS/MS analysis in a randomized order. An Agilent 1100 Capillary LC system (Agilent Technologies, San Jose, CA) and a Q Exactive mass spectrometer (ThermoFisher Scientific Inc.) were used. Peptides were separated on a tip column (75-μm inner diameter × 50 cm) packed with reverse phase beads (1.9 μm/120 Å ReproSil-Pur C18 resin, Dr. Maisch GmbH, Ammerbuch, Germany) using a 90-min gradient from 5 to 30% (v/v) acetonitrile at a 200 nL/min flow rate. 0.1% (v/v) formic acid in water was used as solvent A, and 0.1% FA in 80% acetonitrile was used as solvent B. The MS scan was performed from 300 to 1800 m/z, followed by data-dependent MS/MS scan of the 12 most intense ions, a dynamic exclusion repeat count of two, and repeat exclusion duration of 30 s were used. The resolutions for MS and MS/MS were 70,000 and 17,500, respectively.

### Assessment of the equal-volume strategy

Six dilutions of a single microbiome sample were prepared in triplicate wells and an equal volume was taken from each sample for tryptic digestion and LC-MS/MS analysis. Metaproteomic sample processing and analysis followed the same procedures stated above, and total peptide intensity was calculated. A DC protein concentration assay was also performed with each sample. Linearity between total protein concentration and total peptide intensity quantified by LC-MS/MS was then compared.

### Metaproteomics data analysis

Protein/peptide identification and quantification, taxonomic assignment, and functional annotations were done using the MetaLab software (version 1.1.0) [27]. MetaLab is a software that automates an iterative database search strategy, i.e., MetaPro-IQ [29]. The search was based on a human gut microbial gene catalog containing 9,878,647 sequences from http://meta.genomics.cn/. In MetaLab, a spectral clustering strategy [27] was used for database construction from all raw files, then the peptide and protein lists were generated by applying strict filtering based on a FDR of 0.01, and quantitative information for proteins was obtained with the maxLFQ algorithm on MaxQuant (version 1.5.3.30). Carbamidomethyl (C) was set as a fixed modification and oxidation (M) and N-terminal acetylation (Protein N-term) were set as variable modifications. The matching between runs option was used. Instrument resolution was set as “High-High.”

Total microbiome biomass was estimated for each sample by summing peptide intensities. Taxonomic identification was achieved by assigning peptide sequences to lineage of lowest common ancestor (LCA). The “peptide to taxonomy” database (pep2tax database) was selected for mapping identified peptides to the taxonomic lineages [27]. Bacteria, eukaryota, viruses, and archaea were included in the LCA calculation. Taxonomic biomass was quantified by summing the intensities of the peptides corresponding to each taxon. A Bray-Curtis dissimilarity-based approach [32] was applied for evaluating the variation of genus-level biomass contributions between drug-treated and DMSO control groups. Calculation of the Bray-Curtis distance was performed using the R package “vegan” [33].

The quantified protein groups were first filtered according to the criteria that the protein appears in > 80% of the microbiomes with at least one drug treatment. Then, LFQ protein group intensities of the filtered file were log_2_-transformed and normalized through quotient transformation (*x*/mean) using the R package “clusterSim.” Variance associated with the individual signature was evaluated by PerMANOVA test [31] based on Bray-Curtis dissimilarities [32, 33] using the R package “vegan.” Then, LFQ protein group intensities were processed by a ComBat process [58, 59] using iMetalab.ca [60] to remove possible batch effects between individual microbiomes. Using the ComBat-corrected data, an unsupervised non-linear dimensionality reduction algorithm, t-distributed stochastic neighbor embedding (t-SNE) [34] was then applied to visualize similarities between samples using the R package “Rtsne.” Parameter for the function Rtsne() were, perplexity = 10, max_iter = 1200 (number of iterations), other parameters were set as default. The R function geom_polygon implemented in ggplot2 was used to visualize the t-SNE results.

Functional annotations of protein groups, including COG and KEGG information, were obtained in the MetaLab software. In addition, KEGG ortholog (KO) annotation of protein FASTA sequences was conducted using GhostKOALA (https://www.kegg.jp/ghostkoala/) [61]. Log_2_ fold-change of each drug-treated sample relative to the corresponding DMSO control was calculated using the abundances of proteins annotated to COG categories and COGs. Functional enrichment analysis was performed using the enrichment module on iMetalab.ca through inputting the list of COG functional proteins. Adjusted *p* value cutoff was set at 0.05 for the enrichment analysis. We also visualized all functional responses (including COG, KEGG, NOG, and GO terms) using a R Shiny app, which is available at https://shiny.imetalab.ca/RapidAIM_functional_response/. In this app, fold change of a function between treated and control microbiomes was visualized using boxplots, and statistical significances were calculated using Wilcoxon test.

### Statistical analysis

We examined data distribution on all levels of data, and results indicated non-normal distributions of the dataset (examples shown in Supplementary Figures S12 and S13). Hence, a non-parametric statistical hypothesis test, the Wilcoxon rank sum test, was applied in statistical analyses. For multiple comparisons, *p* values were adjusted using the Benjamini-Hochberg false discovery rate (FDR) procedure [62]. For multivariate analysis, partial least-squares discriminant analyses (PLS-DA) based on ComBat-corrected protein group intensities were performed using MetaboAnalyst (http://www.metaboanalyst.ca/) [63]. PLS-DA model were evaluated by cross-validation of *R*^2^ and *Q*^2^.

### Data visualizations

Box plots, violin plots, hierarchical clustering, 3D scatter plots, heatmaps, PCA, and t-SNE were visualized using R packages ggplot2, gridExtra, scatterplot3d, and pheatmap. Pathway maps were visualized using iPATH 3 (https://pathways.embl.de/) [64] and Pathview Web (https://pathview.uncc.edu/) [65]. Stacked column bars and functional enrichments were visualized on iMetaLab.ca.

## Supplementary information

**Additional file 1: Table S1.** Information of 43 tested compounds. **Table S2.** Significantly shifted species under drug treatment (one-sided Wilcoxon rank sum test, FDR-adjusted *p* < 0.05).

**Additional file 2: Figure S1.** Establishment and step-by-step validation of the microplate-based metaproteomic sample preparation workflow of the RapidAIM assay. **Figure S2.** Assessment of the equal-volume digestion and LC-MS/MS analysis strategy. **Figure S3.** Data quality check of the POC dataset. **Figure S4.** Reproducibility of RapidAIM assay on different levels. **Figure S5.** Case study on microbiome V1’s response to rifaximin. **Figure S6.** Log_2_ fold-change of relative abundance at the genus level in response to each drug compared with the DMSO control. **Figure S7.** Score plots and cross-validations of seven PLS-DA models. **Figure S8.** Log_2_ fold-change of functions at the COG protein level. **Figure S9.** String interaction of COG functional proteins significantly stimulated by diclofenac. **Figure S10.** Response of enzymes along the butyrate production from Acetyl-CoA. **Figure S11.** Phylum-specific functional responses to Berberine. **Figure S12.** Randomly selected LFQ intensities of protein groups showing heavy tailed distribution on the Q-Q plots. **Figure S13.** Randomly selected log_2_-fold changes of COGs showing heavy tailed distribution on the Q-Q plots.

## Data Availability

All raw data from LC-MS/MS have been deposited to the ProteomeXchange Consortium (http://www.proteomexchange.org) via the PRIDE partner repository (dataset identifiers PXD012724 and PXD012725).

## Abbreviations

COG: Clusters of orthologous groups
DMSO: Dimethyl sulfoxide
FDR: False-discovery rate
FOS: Fructooligosaccharide
GABA: Gamma-aminobutyric acid
KEGG: Kyoto Encyclopedia of Genes and Genomes
LC-MS/MS: Liquid chromatography–tandem mass spectrometry
LFQ: Label-free quantification
NSAIDs: Nonsteroidal anti-inflammatory drugs
PCA: Principle component analysis
PLS-DA: Partial least squares discriminant analysis
POC: Proof of concept
VIP: Variable importance in projection
