# Supplementary material for: RapidAIM: a culture- and metaproteomics-based Rapid Assay of Individual Microbiome responses to drugs
Source: Microbiome. 2020 Mar 11;8:33. doi: 10.1186/s40168-020-00806-z (PMC7066843; doi:10.1186/s40168-020-00806-z)
Supplement: Supplementary file 2 — Additional file 2: Figure S1. Establishment and step-by-step validation of the microplate-based metaproteomic sample preparation workflow of the RapidAIM assay. Figure S2. Assessment of the equal-volume digestion and LC-MS/MS analysis strategy. Figure S3. Data quality check of the POC dataset. Figure S4. Reproducibility of RapidAIM assay on different levels. Figure S5. Case study on microbiome V1’s response to rifaximin. Figure S6. Log2 fold-change of relative abundance at the genus level in response to each drug compared with the DMSO control. Figure S7. Score plots and cross-validations of seven PLS-DA models. Figure S8. Log2 fold-change of functions at the COG protein level. Figure S9. String interaction of COG functional proteins significantly stimulated by diclofenac. Figure S10. Response of enzymes along the butyrate production from Acetyl-CoA. Figure S11. Phylum-specific functional responses to Berberine. Figure S12. Randomly selected LFQ intensities of protein groups showing heavy tailed distribution on the Q-Q plots. Figure S13. Randomly selected log2-fold changes of COGs showing heavy tailed distribution on the Q-Q plots. [file 40168_2020_806_MOESM2_ESM.docx]

RapidAIM: A culture- and metaproteomics-based Rapid Assay of Individual Microbiome responses to drugs

Leyuan Li^1^, Zhibin Ning^1^, Xu Zhang^1^, Janice Mayne^1^, Kai Cheng^1^, Alain Stintzi*^1^, Daniel Figeys*^1,2,3^

^1^ Department of Biochemistry, Microbiology and Immunology, Ottawa Institute of Systems Biology, Faculty of Medicine, University of Ottawa, Ottawa, Canada

^2^ Department of Chemistry and Biomolecular Sciences, University of Ottawa, Ottawa, Canada

^3^ Canadian Institute for Advanced Research, Toronto, Canada

* Corresponding Authors, Email: [dfigeys@uottawa.ca](mailto:dfigeys@uottawa.ca) (DF), and [astintzi@uottawa.ca](mailto:astintzi@uottawa.ca) (AS)

Supplementary figures

Figure S1. Establishment and step-by-step validation of the microplate-based metaproteomic sample preparation workflow of the RapidAIM assay

Figure S2. Assessment of the equal-volume digestion and LC-MS/MS analysis strategy

Figure S3. Data quality check of the POC dataset

Figure S4. Reproducibility of RapidAIM assay on different levels

Figure S5. Case study on microbiome V1’s response to rifaximin

Figure S6. Log_2_ fold-change of relative abundance at the genus level in response to each drug compared with the DMSO control

Figure S7. Score plots and cross-validations of seven PLS-DA models

Figure S8. Log_2_ fold-change of functions at the COG protein level

Figure S9. String interaction of COG functional proteins significantly stimulated by diclofenac

Figure S10. Response of enzymes along the butyrate production from Acetyl-CoA

Figure S11. Phylum-specific functional responses to Berberine

Figure S12. Randomly selected LFQ intensities of protein groups showing heavy tailed distribution on the Q-Q plots

Figure S13. Randomly selected log_2_-fold changes of COGs showing heavy tailed distribution on the Q-Q plots

**Supplementary Figure S1**


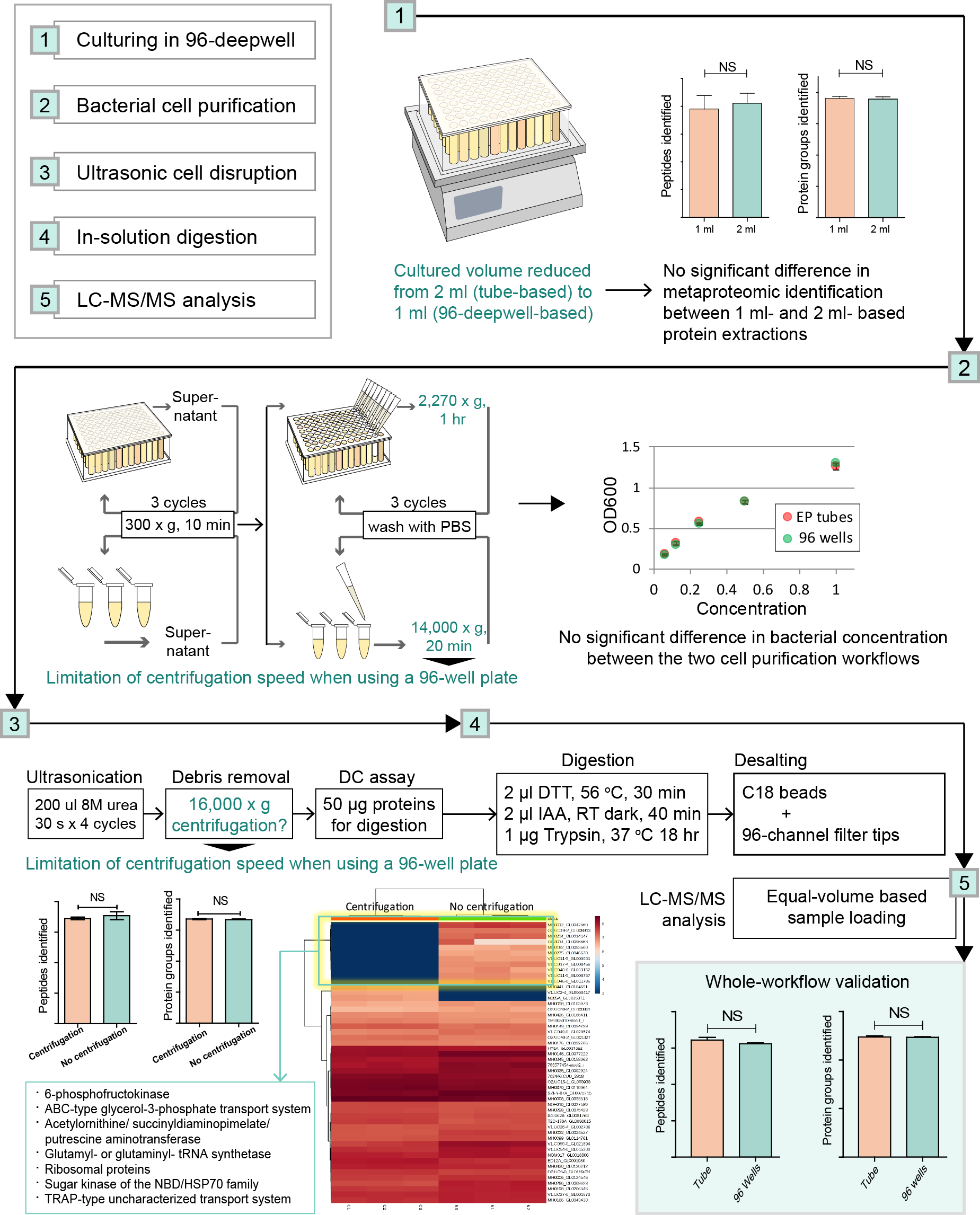


**Figure S1. Establishment and step-by-step validation of the microplate-based metaproteomic sample preparation workflow of the RapidAIM assay.**

After culturing in a 96-well deepwell plate **[1]**, the bacterial cells were washed with PBS **[2]**, then disrupted with four cycles of 30 s ultrasonication **[3]**. Protein concentration was measured in the DMSO control sample using a DC protein assay. This concentration was used to calculate a volume equivalent to 50 µg proteins in the control sample. This volume was taken from every sample and digested with trypsin **[4]**, then desalted with a panel of 96 filter tips packed with C18 beads and equal volumes of each sample were analyzed by LC-MS/MS **[5]**.

Due to a few differences in the metaproteomic procedure compared with tube-based protocols, we examined the effect of protocol differences on sample quality step-by-step. In previously published protocols, samples were cultured in 2 ml culture tubes^1,2^. In step **[1]**, culturing samples in a 96-well plate reduced the sample size to 1 ml. No significant difference was shown by *t*-test in peptide and protein group identification numbers between protein extractions from 1 ml and 2 ml samples. For step **[2]**, the 96-deepwell plates limited centrifugation speed to 2,270 g (versus 14,000 g in the original protocol), so we extended the centrifugation time from 20 min to 1 hour and tested whether bacterial concentration was affected by the altered centrifugation process^1,3^. Concentration of purified bacterial cells were compared by OD_600_ after being re-suspended in 1 ml, 2 ml, 4 ml, 8 ml, and 16 ml PBS. No significant difference in OD_600_ reads was observed between the two cell purification protocols. In step **[3]**, the original protocol used a high-speed centrifugation (16,000 g) to remove cell debris after the ultrasonication. Due to the limitation of centrifugation speed when using a microplate, we compared metaproteomic profiles of the sample when digested with or without cell debris removal. No significant differences were found in the number of protein identifications. In terms of differentially identified proteins, we found that the samples without cell debris removal resulted in more identifications of cell-membrane proteins such as the ABC-type transport systems, as well as cytoskeleton-related proteins, such as the translation elongation factor EF-Tu^4,5^, 6-phosphofructokinase^6^, and ribosomes^7^. Therefore, we infer that eliminating the centrifugation process could reduce the removal of cytoskeleton and cell membrane proteins. For step **[4]**, no validation was necessary as the same type of filter tips were used in both protocols. Finally, we performed a whole-workflow comparison of the metaproteomic outcomes between traditional tube-based and 96-well-based processes. The microplate-based metaproteomic workflow showed no statistically significant difference in peptide and protein identification compared with the tube-based workflow.

**Supplementary Figure S2**


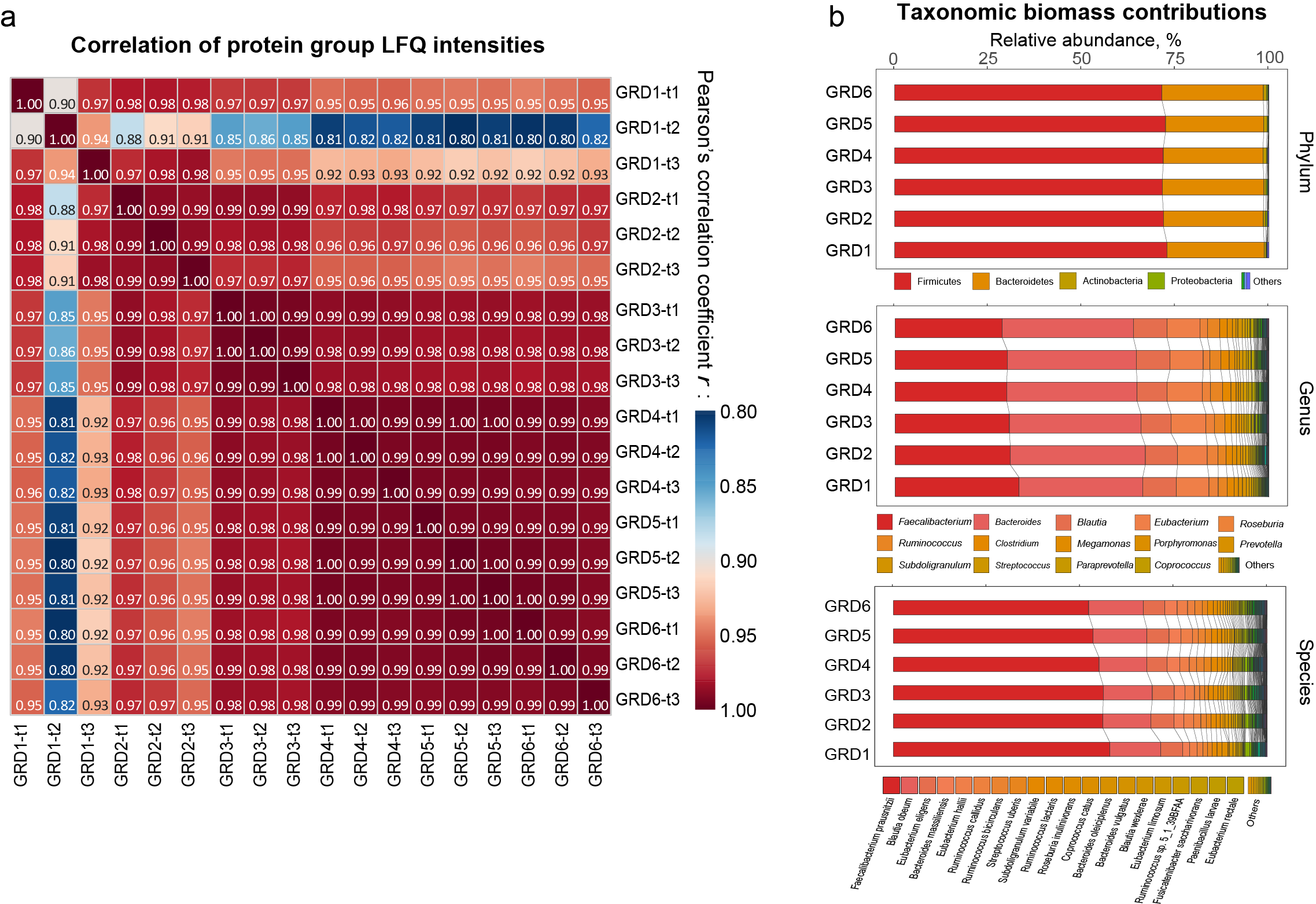


**Figure S2. Assessment of the equal-volume digestion and LC-MS/MS analysis strategy.**

(**a**) In triplicates, six dilutions of a single microbiome sample were subjected to this equal-volume based analysis.

The LFQ intensities of protein groups showed Pearson’s correlation coefficient *r* > 0.95 between most dilutions, but a lower *r* was seen in the samples with the lowest concentration. (**b**) Comparison of taxonomic biomass contributions on different levels (summed peptide intensity assigned to different taxa) among diluted groups suggested very low level of bias. GRD1-6 are six different dilution gradients (protein concentration shown in Figure 1b), and t1-t3 are technical replicates of the same conditions.

**Supplementary Figure S3**

**
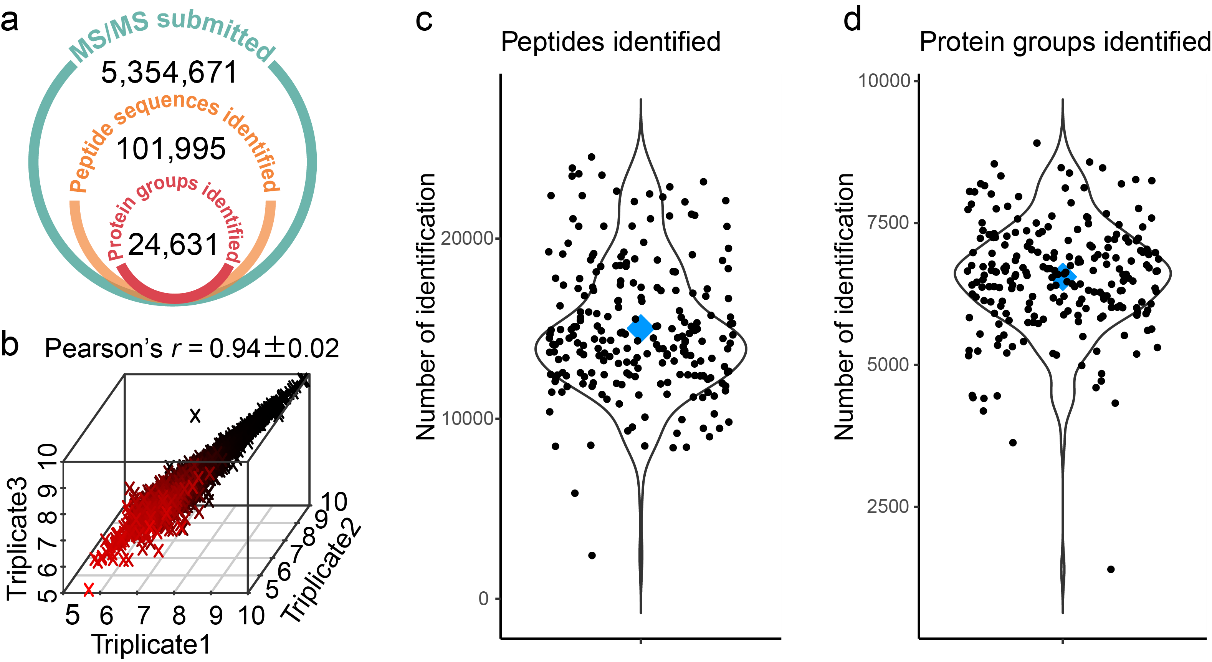
**

**Figure S3. Data quality check of the POC dataset.**

(a) Numbers of MS/MS submitted, peptide sequence and protein group identifications in the POC dataset. (b) Analysis of three technical replicates with RapidAIM showing high Pearson’s correlation. (c) Peptide identification on an individual sample metrics. (d) Protein group identification on an individual sample metrics. In (c) and (d), black dots are individual samples, and blue diamonds represent the average value of the POC dataset.

**Supplementary Figure S4**


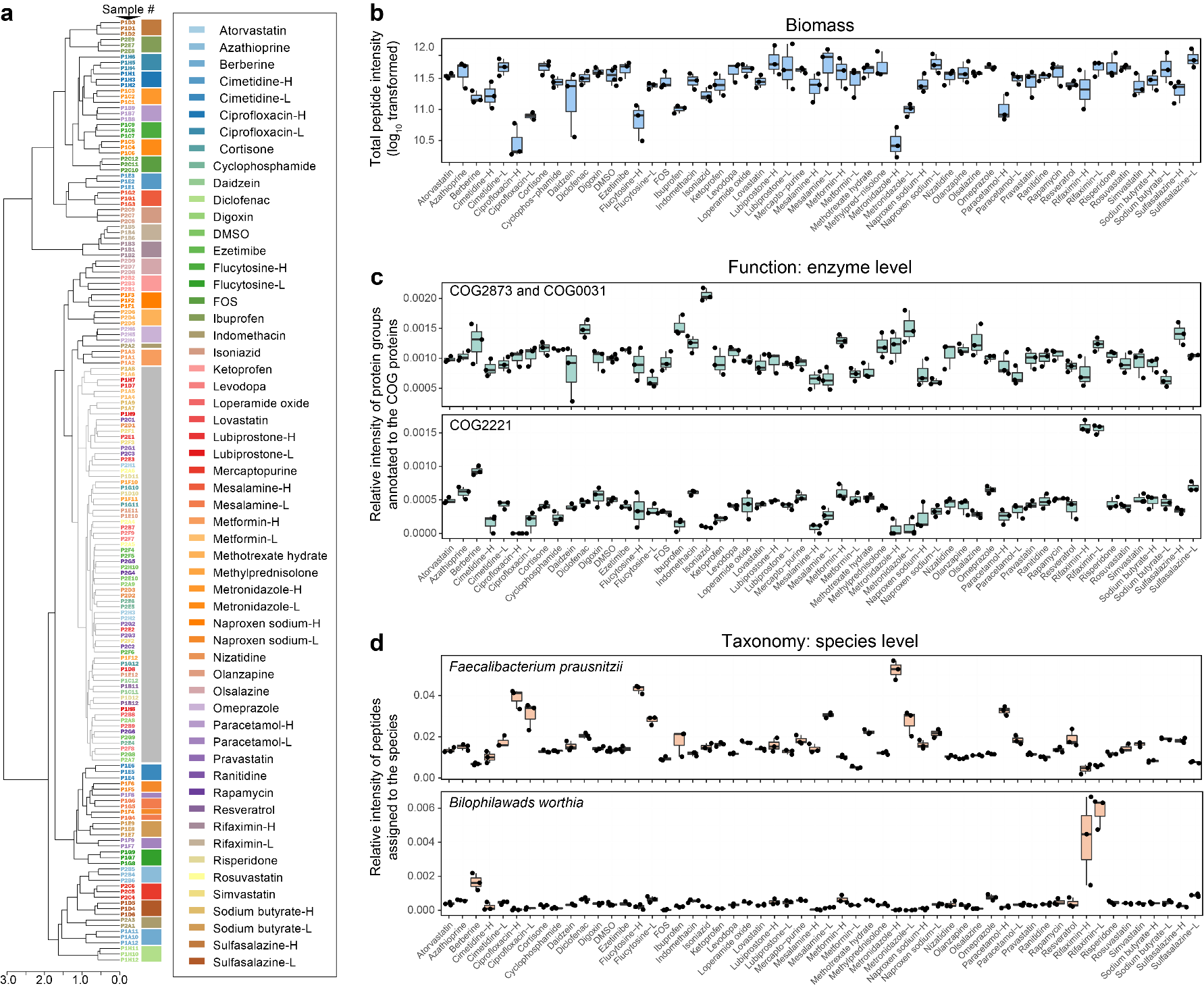


**Figure S4. Reproducibility of RapidAIM assay on different levels.**

(**a**) Clustering using protein group composition information showed that triplicates of drug treatments were closely clustered. A hierarchical tree was generated based on Pearson’s correlation coefficient. The cluster corresponding to the gray box contained DMSO control samples; samples in this cluster indicated drugs that had very weak effects on the microbiome. (**b**) Box chart suggesting that biomass effects by drugs are highly reproducible. (**c**) Examples showing reproducible functional responses to drugs at the enzyme level. COC0031 and COG2873 are cysteine synthase and O-acetylhomoserine/O-acetylserine sulfhydrylase (pyridoxal phosphate-dependent), respectively; both enzymes are involved in the conversion of sulfide to L-cysteine^8^. COG2221 is dissimilatory sulfite reductase (desulfoviridin), alpha and beta subunits; this enzyme reduces sulfite to sulfide^9^. (**d**) Examples showing reproducible taxonomic responses to drugs at the species level. *F. prausnitzii* is a ubiquitous bacterium of the intestinal microbiota^10^; *B. worthia* is a taurine-degrading bacterium which can reduce sulfite to sulfide by dissimilatory sulfite reductase^9^. COG2221 and *B. worthia* show a correlation in their response to different drugs. Box spans interquartile range (25th to 75th percentile), and line within box denotes median.

**Supplementary Figure S5**


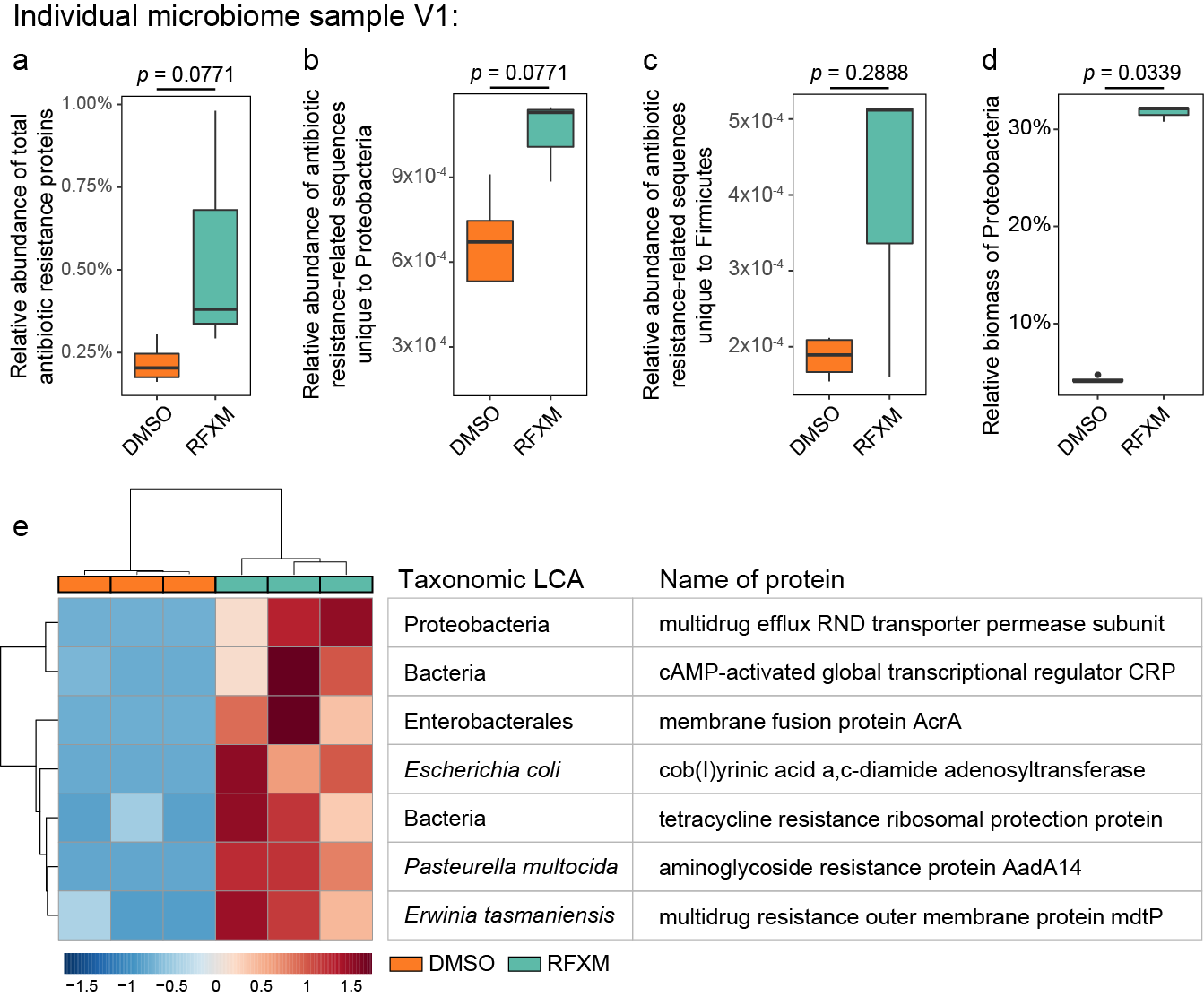


**Figure S5. Case study on microbiome V1’s response to rifaximin (RFXM).** We have shown that the antibiotic rifaximin did not show overall biomass inhibition on microbiome sample V1 (Figure 2a and Supplementary Figure S3b). We examined whether the expressions of antibiotic resistance proteins were affected. Briefly, the LC-MS/MS raw files were specifically searched against the Structured Antibiotic Resistance Genes (SARG) database 48 which highlighted 118 antibiotic resistance protein groups across the dataset. (**a**) We found an increase in relative abundance of total antibiotic resistance proteins in microbiome V1 in response to rifaximin. (**b and c**) Particularly, antibiotic resistance peptide sequences unique to Proteobacteria and Firmicutes were increased. (**d**) Moreover, despite no significant change in total microbiome biomass in V1, a significant 6.5-fold increase in the relative biomass of Proteobacteria in the whole microbial community was observed. (**e**) Non-parametric test resulted in seven significantly increased antibiotic resistance protein groups (FDR-adjusted p value<0.05). These protein groups belonged predominantly to Proteobacteria (5 out of 7). Increase of Proteobacteria is associated with dysbiosis in gut microbiota 49. These together suggested a potential risk of rifaximin administration in individual V1. (*p* values were based on two-sided Wilcoxon test; box spans interquartile range (25th to 75th percentile), and line within box denotes median.

**Supplementary Figure S6**


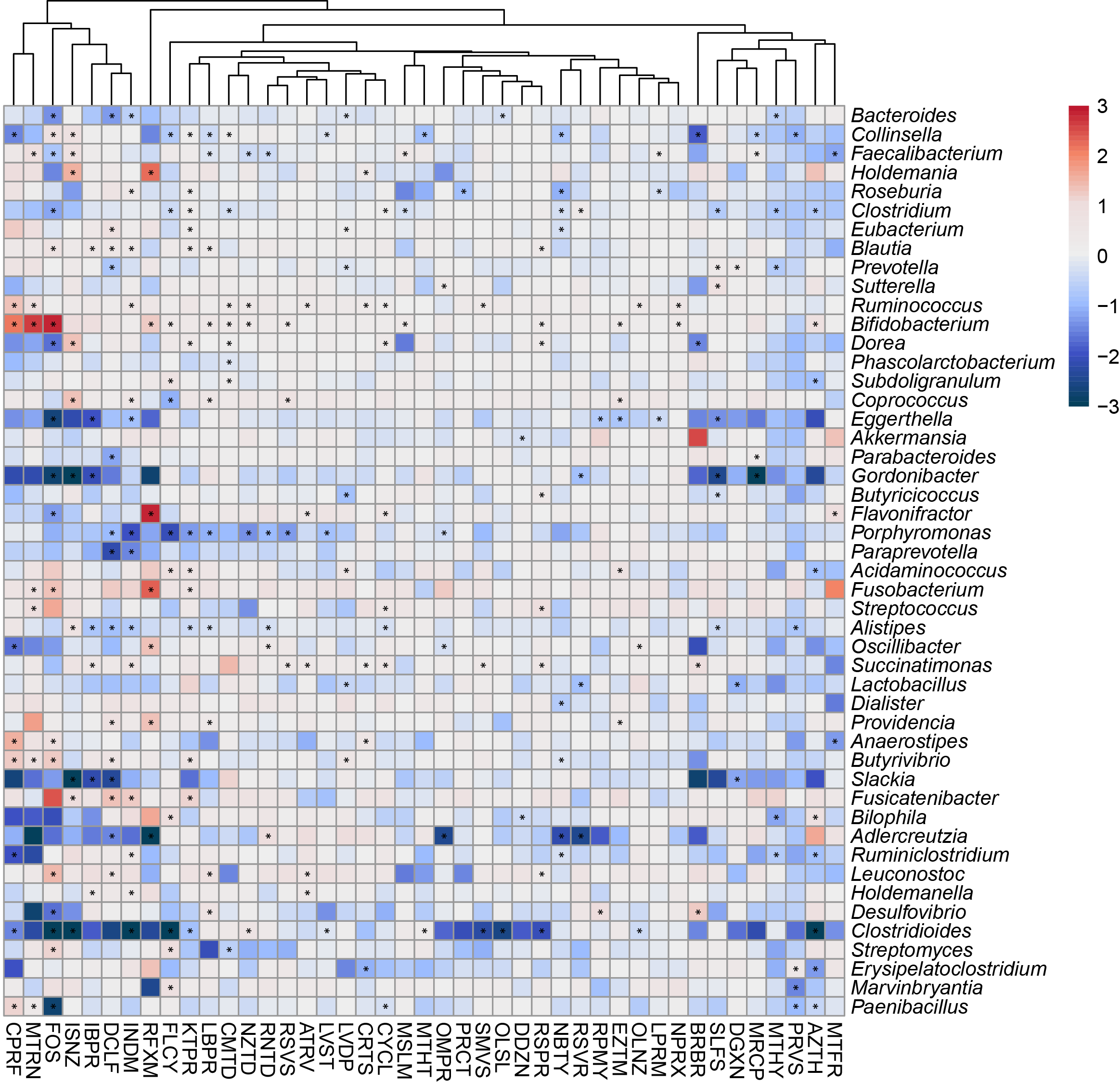


**Figure S6. Log_2_ fold-change of relative abundance at the genus level in response to each drug compared with the DMSO control.** Genera that existed in ≥80% of the volunteers are shown. Star (*) indicate significantly changed bacterial abundance by Wilcoxon test, *p* <0.05..

**Supplementary Figure S7**


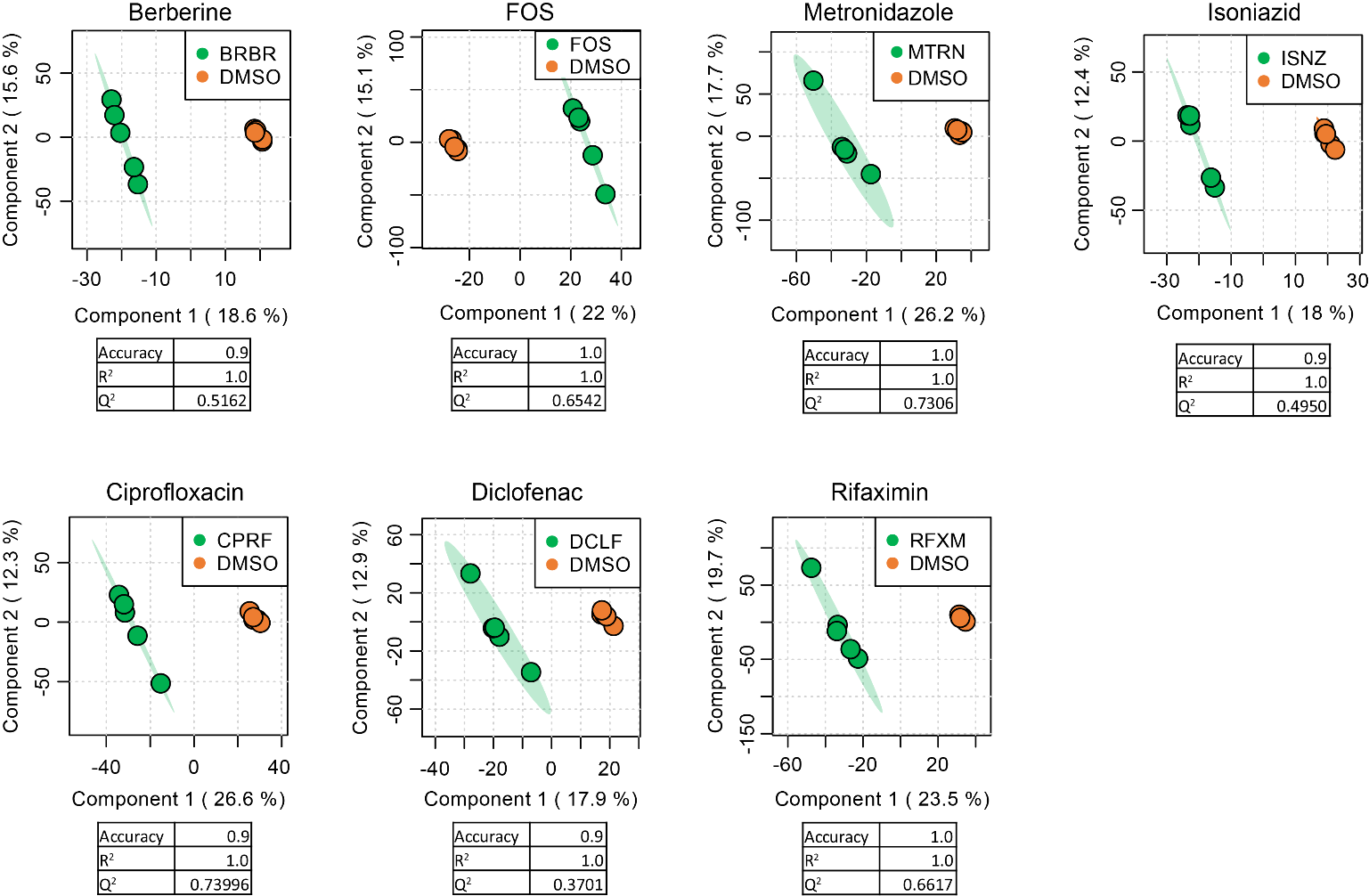


**Figure S7. Score plots and cross-validations of seven PLS-DA models**

PLS-DA models of microbiomes responses to each compound were established using MetaboAnalyst 4.0. PLS-DA model qualities were assessed through cross-validation, and accuracy, R^2^ and Q^2^ were given for each model. Seven compounds were found with valid PLS-DA models distinguishing the effect of the compound from the DMSO control.

**Supplementary Figure S8**


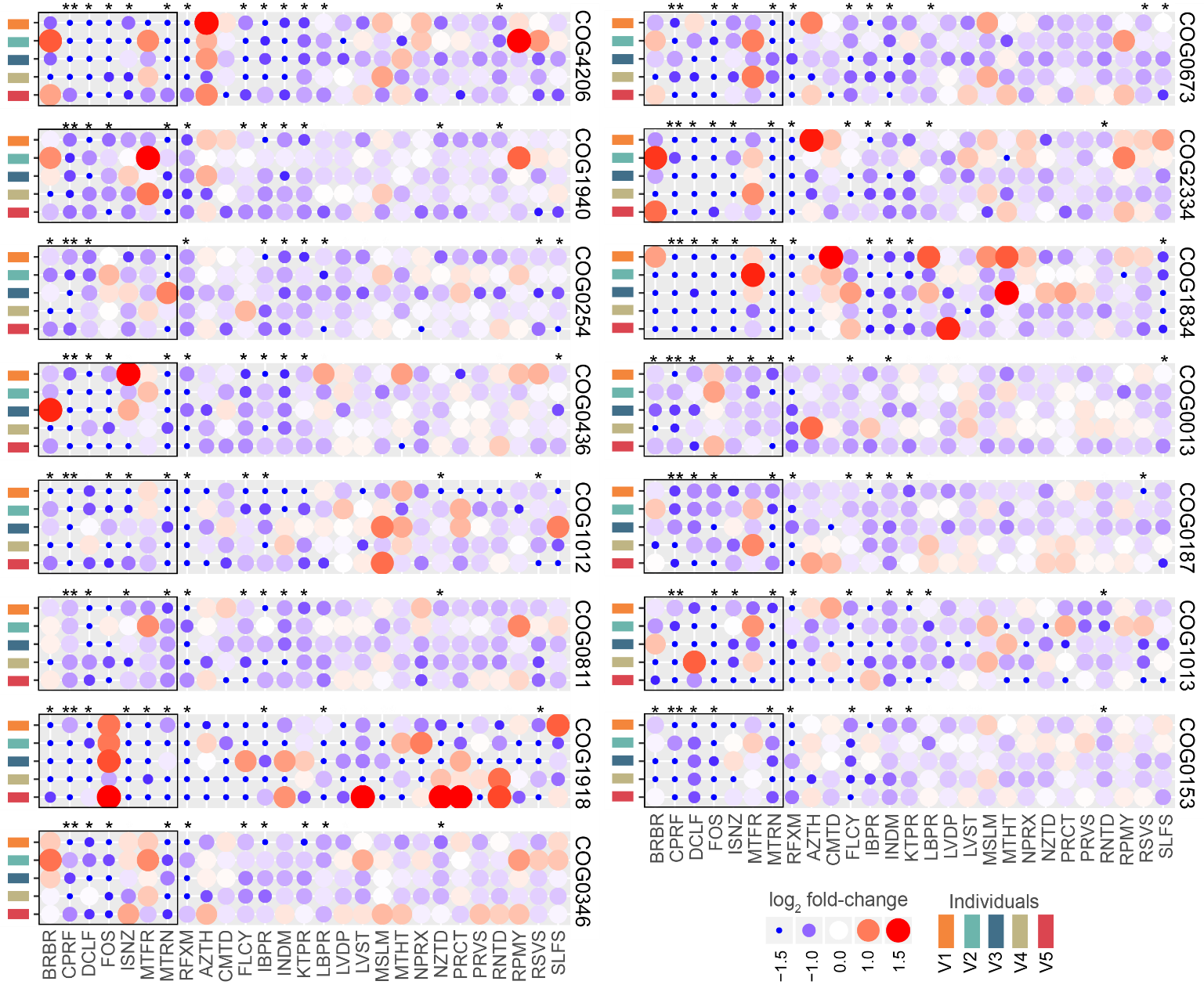


**Figure S8. Log_2_ fold-change of functions at the COG protein level**

On the COG functional protein level, 535 COGs were significantly decreased by at least one drug treatment. The 15 COGs that were affected by ≥ 10 compounds are shown in this figure. Statistical significance was evaluated by one-sided Wilcoxon rank sum test, FDR-adjusted *p*-values: *, *p* < 0.05; **, *p* < 0.01.

**Supplementary Figure S9**

**
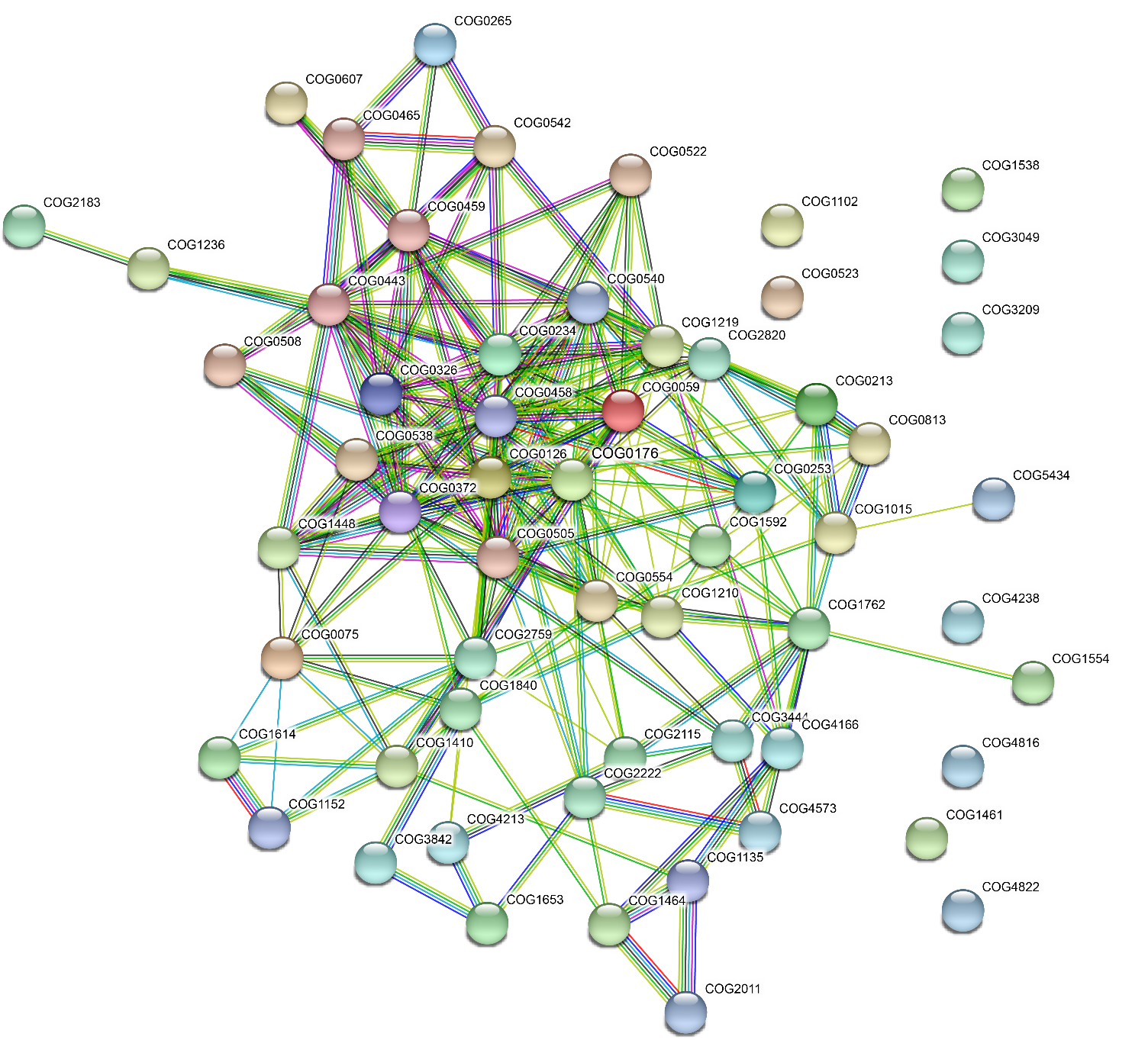
**

**Figure S9. String interaction of COG functional proteins significantly stimulated by diclofenac**

**Supplementary Figure S10**


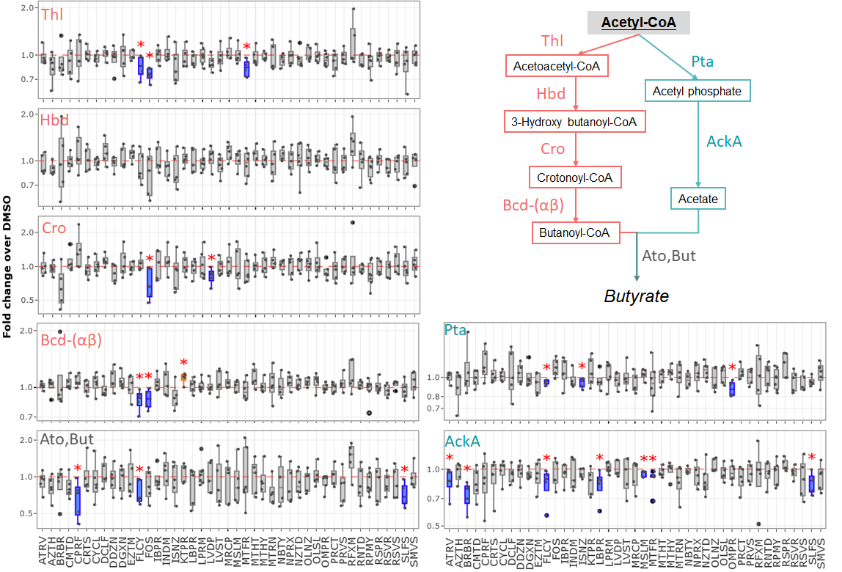
\

**Figure S10. Response of enzymes along the butyrate production from Acetyl-CoA.**

* denotes p < 0.05 by Wilcoxon test. boxplots of all functional responses are available at <https://shiny.imetalab.ca/RapidAIM_functional_response/>.

**Supplementary Figure S11**


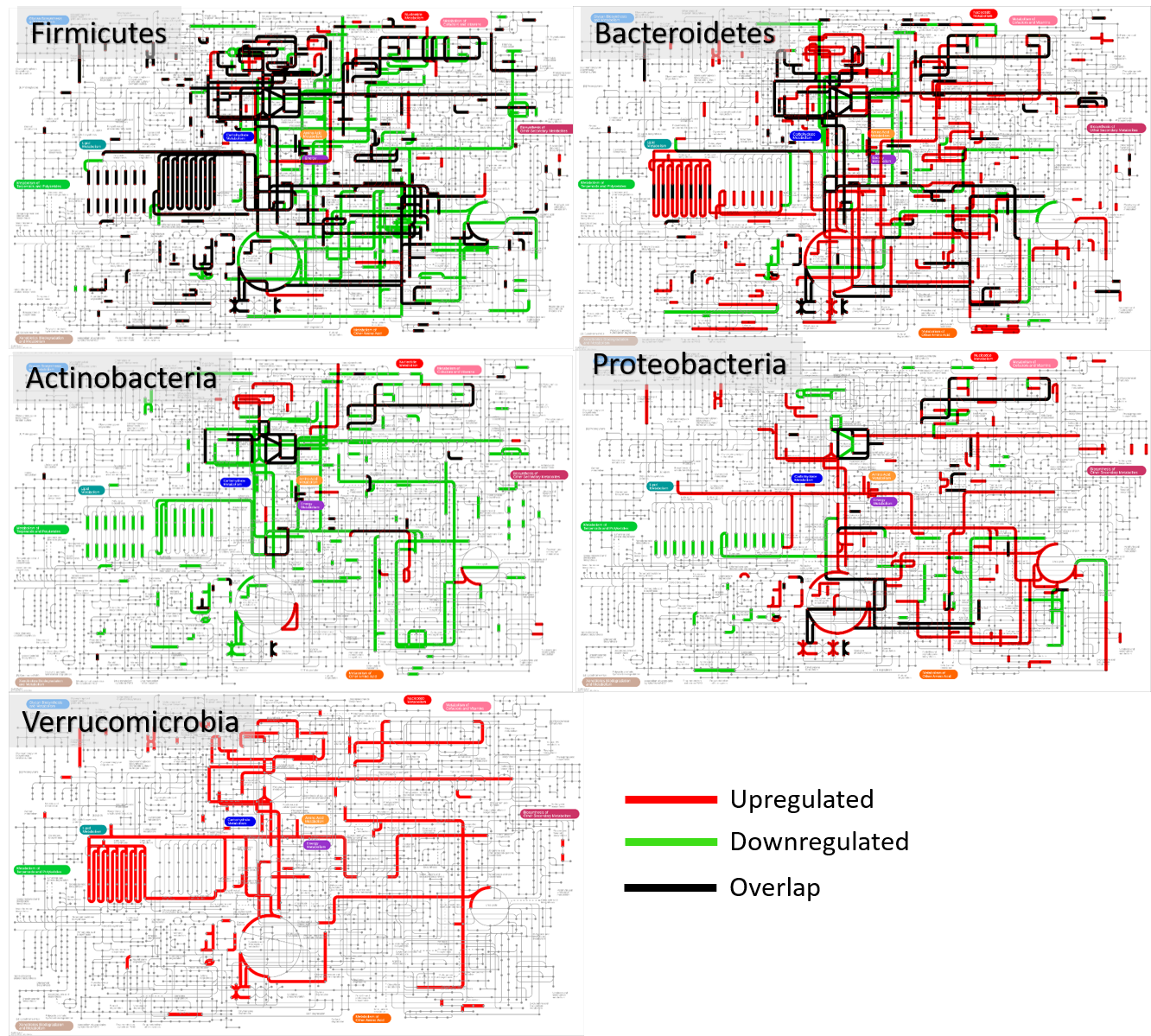


**Figure S11. Phylum-specific functional responses to Berberine**

Protein groups with VIP scores of >1 were annotated to phyla and COGs. Up- and down-regulated (red and green lines) COGs corresponding to different phyla were illustrated on a metabolic pathway map using iPath 3. Pathway maps for each phylum were combined, and overlapped pathways were shown in black lines. Our data suggest that different phyla responded differently at a functional pathway level.

**Supplementary Figure S12**

**
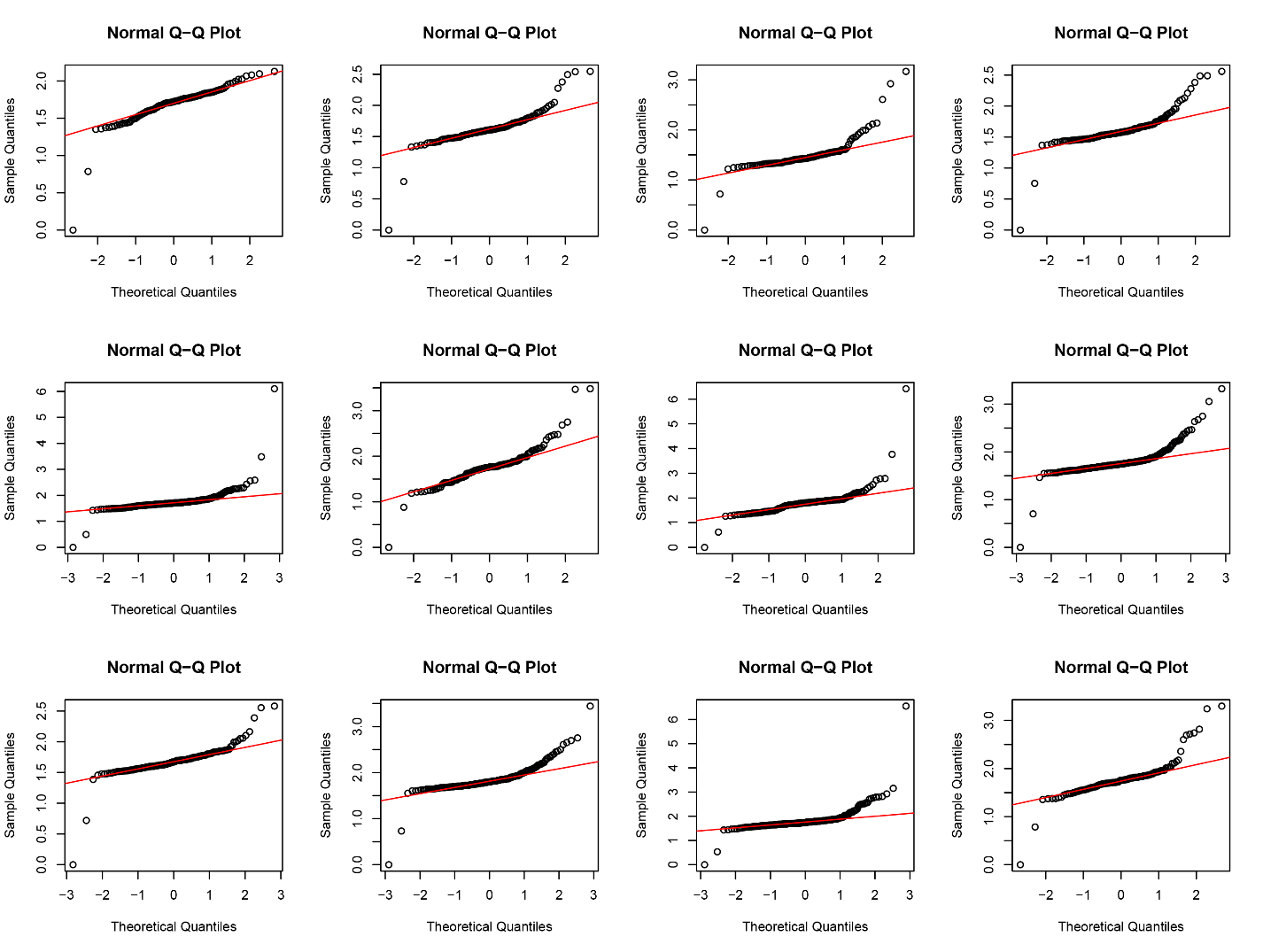
**

**Figure S12. Randomly selected LFQ intensities of protein groups showing heavy tailed distribution on the Q-Q plots.**

**Supplementary Figure S13**

**
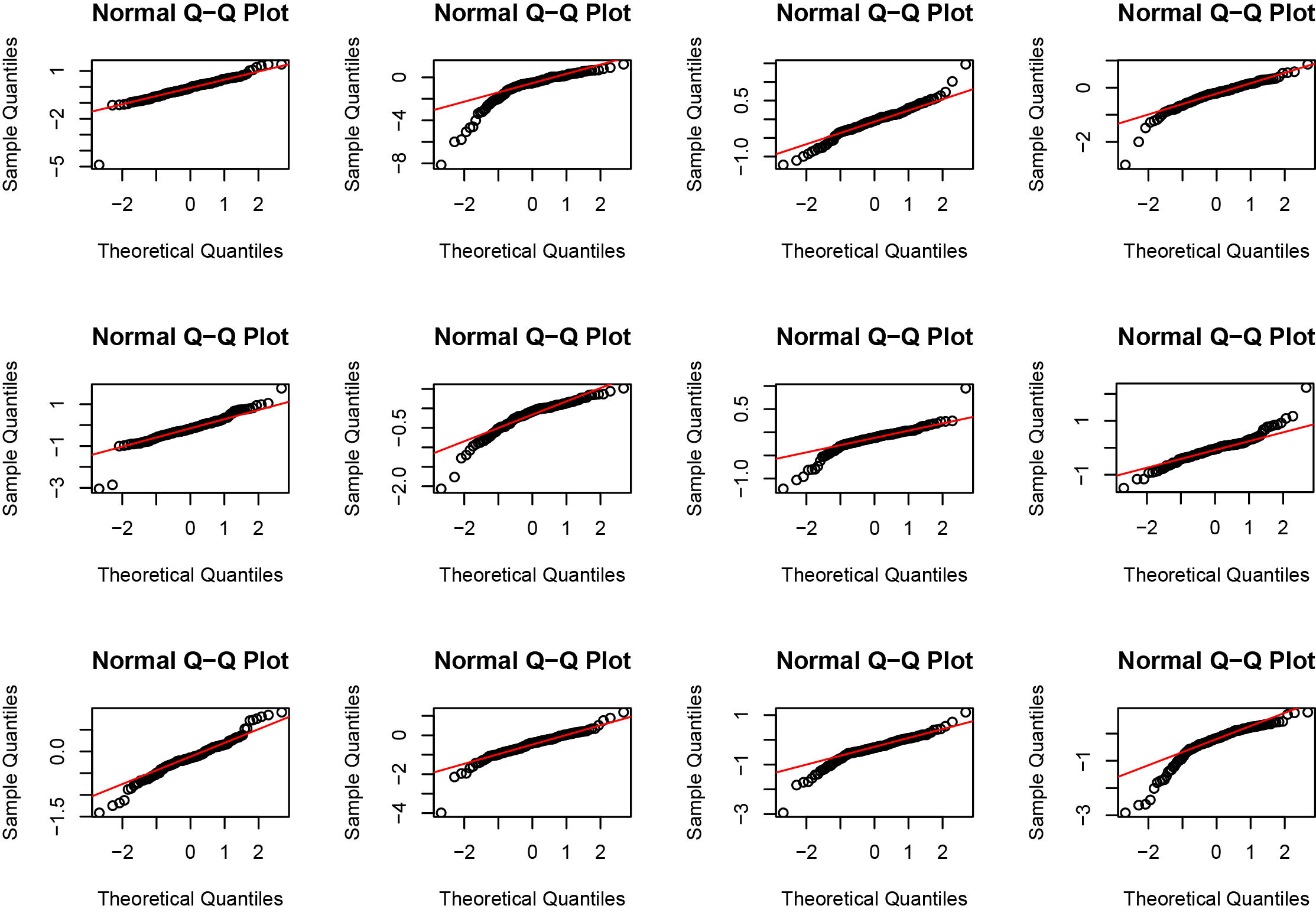
**

**Figure S13. Randomly selected log_2_-fold changes of COGs showing heavy tailed distribution on the Q-Q plots.**

**References**

1. Li, L.*, et al.* Evaluating *in vitro* culture medium of gut microbiome with orthogonal experimental design and a metaproteomics approach. *Journal of Proteome Research* **17**, 154-163 (2018).

2. Zhang, X.*, et al.* *In vitro* metabolic labeling of intestinal microbiota for quantitative metaproteomics. *Analytical Chemistry* **88**, 6120-6125 (2016).

3. Zhang, X.*, et al.* Assessing the impact of protein extraction methods for human gut metaproteomics. *Journal of Proteomics* **180**, 120-127 (2018).

4. Defeu Soufo, H.J., Reimold, C., Breddermann, H., Mannherz, H.G. & Graumann, P.L. Translation elongation factor EF-Tu modulates filament formation of actin-like MreB protein *in vitro*. *Journal of Molecular Biology* **427**, 1715-1727 (2015).

5. Mayer, F. Cytoskeletal elements in bacteria *Mycoplasma pneumoniae*, *Thermoanaerobacterium* sp., and *Escherichia coli* as revealed by electron microscopy. *Journal of Molecular Microbiology and Biotechnology* **11**, 228-243 (2006).

6. Vértessy, B.G., Orosz, F., Kovács, J. & Ovádi, J. Alternative binding of two sequential glycolytic enzymes to microtubules: molecular studies in the phosphofructokinase/aldolase/microtubule system. *Journal of Biological Chemistry* **272**, 25542-25546 (1997).

7. Mayer, F. Cytoskeletons in prokaryotes. *Cell Biology International* **27**, 429-438 (2013).

8. Lithgow, J.K., Hayhurst, E.J., Cohen, G., Aharonowitz, Y. & Foster, S.J. Role of a cysteine synthase in *Staphylococcus aureus*. *Journal of Bacteriology* **186**, 1579 (2004).

9. Laue, H., Friedrich, M., Ruff, J. & Cook, A.M. Dissimilatory sulfite reductase (desulfoviridin) of the taurine-degrading, non-sulfate-reducing bacterium *Bilophila wadsworthia* RZATAU contains a fused DsrB-DsrD subunit. *Journal of Bacteriology* **183**, 1727 (2001).

10. Miquel, S.*, et al.* *Faecalibacterium prausnitzii* and human intestinal health. *Current Opinion in Microbiology* **16**, 255-261 (2013).
